# Health-seeking behavior and its determinants for non-communicable diseases in India - a systematic review and meta-analysis

**DOI:** 10.3389/fpubh.2025.1580824

**Published:** 2025-06-11

**Authors:** Madhumitha Haridoss, Dhruva Nandi, Raji Rajesh Lenin, Shiny P. John, V. V. Anantharaman, Rajiv Janardhanan

**Affiliations:** ^1^Division of Medical Research, Faculty of Medicine and Health Sciences, SRM Medical College Hospital and Research Centre, SRM Institute of Science and Technology, Kattankulathur, India; ^2^Department of Community Medicine, Faculty of Medicine and Health Sciences, SRM Medical College Hospital and Research Centre, SRM Institute of Science and Technology, Kattankulathur, India

**Keywords:** health-seeking behavior, healthcare access, non-communicable diseases, NPCDCS, India, systematic review, meta-analysis

## Abstract

**Introduction:**

India faces a growing burden of non-communicable diseases (NCDs), particularly diabetes, cardiovascular conditions, and cancer, straining the healthcare system. Given the urgent need for prevention and management, a systematic review and meta-analysis (SRMA) of health-seeking behaviors for NCDs is essential to guide targeted interventions to improve health outcomes.

**Methods:**

The SRMA protocol was registered in PROSPERO (CRD42023476381) and conducted adhering to the Preferred Reporting Items of Systematic reviews and Meta-Analysis (PRISMA) 2020 guidelines. PubMed-Medline and Scopus databases were searched from inception to October 27, 2023. Eligible studies focused on adults (>18 years) with NCDs covered under the National Programme for prevention and Control of Cancer, Diabetes, Cardiovascular Diseases and stroke (NPCDCS). Data extraction and risk of bias assessment were conducted using predefined criteria. Meta-analysis of quantitative data was performed using DerSimonian and Laird random-effect model.

**Results:**

From 2,917 identified studies, 64 were included in the SRMA, with 40 suitable for meta-analysis. The meta-analysis revealed that 72.72% (95% CI 59.48–85.97%, *I*^2^ = 99.97%) of individuals sought treatment for existing health conditions, with 73.09% (95% CI 54.01–92.16%, *I*^2^ = 99.18%) preferring allopathy, compared to 8.89% (95% CI 5.56–12.22%, *I*^2^ = 86.73%) preferring Alternative medicine with a significant heterogeneity. Major barriers to seeking treatment included illness not considered serious [0.4785 (95% CI 0.4556–0.5013)] and financial constraints [0.3263 (95% CI 0.1457–0.5069)], with delays in cancer treatment attributed to lack of disease awareness [0.5091 (95% CI 0.0294–0.9888)] and painlessness [0.4502 (95% CI 0.3312–0.5692)]. Private healthcare facilities (51.26, 95% CI 42.85–59.67%) were preferred over government facilities (33.78, 95% CI 28.10–39.45%).

**Conclusion:**

This SRMA provide a comprehensive overview of health-seeking behavior for NCDs in India. The findings underscore the complex interplay of socioeconomic, cultural, and systemic factors influencing healthcare access and outcomes. Targeted interventions addressing barriers identified in this review are imperative for improving public health and reducing the burden of NCDs in India.

**Systematic review registration:**

https://www.crd.york.ac.uk/PROSPERO/view/CRD42023476381.

## Introduction

India is experiencing a rising burden of non-communicable diseases (NCDs) in the recent decades along with unfinished agenda of infectious diseases with a non-communicable sequelae (1, 2). Among the major NCDs, cardiovascular disease and stroke account for the significant reduction in the number of productive years and increased premature deaths (3). According to the study report of the India State-Level Disease Burden Initiative which is supported by Indian Council of Medical Research (ICMR), a 23.9% increase in the proportions of deaths due to NCDs from 1990 to 2016 was recorded (4). Additionally, a 3.7% increase in Disability associated life years (DALYs) was observed for the cardiovascular diseases during the same period (4).

The increasing burden of NCDs, places a significant strain on the healthcare system, highlighting the need for niche specific prevention and management strategies. The National Programme for Prevention and Control of Cancer, Diabetes, Cardiovascular Diseases and Stroke (NPCDCS) implemented in India since 2010 is the flagship national program for prevention and control of major NCDs (5). Recognizing health as a fundamental right and understanding the health-seeking behavior among a population is pivotal not only for strengthening niche specific public health surveillance systems, thereby enhancing the health outcomes (6).

Health-seeking behavior encompasses a broad spectrum of choices and actions taken by individuals to enhance, sustain, or ameliorate their health status. Over the years, several studies have examined the health seeking behavior and the barriers to affordability and accessibility of healthcare among the Indian population (7–10). We believe, to the best of our knowledge this Systematic Review and Meta-analysis (SRMA) is a pioneering effort to explore the patterns and processes associated with the health-seeking behavior of the Indian populace. To this end we have conducted a SRMA of studies focusing on the health-seeking behaviors of individuals afflicted with NCDs across the Indian subcontinent. The primary objective of this systematic review and meta-analysis is to synthesize available evidence on the patterns and determinants of health-seeking behavior among individuals with non-communicable diseases (NCDS) in India. Specifically, this review aims to (i) Estimate the prevalence of individuals with NCDs seeking treatment in various settings across India. (ii) Examine the patterns of healthcare utilization, particularly the preference between public and private healthcare providers. (iii) Assess the preferred system of medicine, including the choice between allopathic and AYUSH-based treatments. (iv) Identify and analyse factors associated with health-seeking behavior, such as age, gender, education, socioeconomic status, place of residence, and awareness of disease. (v) Explore the reasons for not seeking treatment and delay in seeking treatment among individuals diagnosed with or exhibiting symptoms of NCDs. Findings of this SRMA will hopefully form the foundation for targeted interventions aimed at improving health outcomes and strengthening public health programs across the Indian subcontinent.

## Methods

### Search strategy and selection criteria

The SRMA protocol was registered in PROSPERO (CRD42023476381) and conducted adhering to the Preferred Reporting Items of Systematic reviews and Meta-Analysis (PRISMA) 2020 guidelines (11). We searched PubMed-Medline and Scopus databases for studies related to health-seeking behavior for NCDs in India from its inception till October 27, 2023. The search terms were identified based on the PEO approach, i.e., P (Population)- Human participants of any age, E (Exposure) -Non-communicable diseases, O (Outcome)- Health-seeking behaviors and S (Settings)- India. Both free text words and MESH terms were wherever necessary. A sensitivity and precision maximizing strategy was adopted to identify relevant studies. The detailed search strategy is presented in Supplementary Tables S1, S2. Inclusion criteria comprises of studies on adults (>18 years) exposed to NCDs under NPCDCS, included diabetes and its complications, hypertension, cardiovascular diseases, stroke, cancer, metabolic associated fatty liver, and chronic obstructive pulmonary disease. Studies assessing health-seeking behaviors, such as seeking treatment, type of healthcare facility visited, lifestyle modifications, and preventive screenings, were included. Studies on communicable diseases, other NCDs (mental health problems, neurodegenerative diseases, orthopedic disorders, and autoimmune diseases), qualitative studies on health-seeking behaviors, and HPV vaccine acceptability studies were excluded. Two reviewers (MH and DN) independently assessed the title, and abstract (TiAb) of the studies based on the eligibility criteria using Rayyan, a web-based tool designed to facilitate systematic review screening. Although Rayyan offers artificial intelligence (AI)-assisted features to expedite the screening process, these features were not utilized in this review. All screening decisions were made manually by the reviewers to ensure methodological rigor and consistency (12). Full text of the studies which passed the TiAb screening were reviewed. Any discrepancies in the decision (inclusion or exclusion) were solved by mutual consensus (Table 1).

**Table 1:** Studies meeting inclusion criteria.

S. No	Study	Study design	Sample size	Disease condition	Location	Setting	Health seeking behavior
1	Kishore et al. (50)	Cross-sectional	98	Diabetes	Barwala and adjoining Pooth Khurd village, New Delhi	Facility	Treatment-seeking
2	Mentock et al. (51)	Cross-sectional	204	Diabetes	Mangaluru, Karnataka	Facility	Treatment-seeking
3	Mishra et al. (42)	Cross-sectional	207	Diabetes	Bhubaneswar, Odisha	Facility	Treatment-seeking
4	Nimesh et al. (18)	Cross-sectional	60	Diabetes	Bhopal, Madhya Pradesh	Community	Treatment-seeking
5	Shukla et al. (19)	Cross-sectional	376	Diabetic retinopathy	11 cities of 9 states in India (Ahmedabad, Bengaluru, Bhubaneswar, Chennai, Delhi, Hyderabad, Jaipur, Kolkata, Mumbai, Pune, Surat)	Facility	Treatment-seeking
6	Srinivas et al. (20)	Cross-sectional	143	Diabetes	Thiruvannamalai District, Tamil Nadu	Community	Treatment-seeking
7	Ahamed et al. (39)	Cross-sectional	457	Self-reported chronic illness mainly diabetes and hypertension	West Bengal	Community	Treatment-seeking
8	Bhojani et al. (52)	Cross-sectional	3,844	Self-reported chronic illness (separately addressing) diabetes and hypertension	Kadugondanahalli (KG Halli), Karnataka	Community	Treatment-seeking
			1,760	Diabetes			
			2,756	Hypertension			
9	Chauhan et al. (53)	Longitudinal	29,443	Diabetes and hypertension	35 states and union territories (except Sikkim)	Community	Treatment-seeking
			8,944	Diabetes			
			20,499	Hypertension			
10	Joshi et al. (54)	Cross-sectional	166	Non-communicable diseases	Punjab	Facility	Treatment-seeking
11	Joshi et al. (40)	Cross-sectional	200	Morbidity profile including diabetes and hypertension	Chandigarh City and Haryana	Community	Treatment-seeking
12	Kanungo et al. (41)	Cross-sectional	43,999	Non-communicable diseases	Malda District, West Bengal	Community	Treatment-seeking
			761	Hypertension			
			374	Diabetes			
			194	Cardiovascular disease			
			67	Cancer			
13	Kusuma et al. (55)	Cross-sectional	15,218	Self-reported chronic illness mainly diabetes and hypertension	NCT, New Delhi	Community	Treatment-seeking
14	Sarkar et al. (56)	Cross-sectional	270	Self-reported chronic illness most commonly diabetes and hypertension	Bhubaneswar, Odisha	Community	Treatment-seeking
15	Singh et al. (57)	Cross-sectional	660	Non-communicable diseases	Punjab	Community	Treatment-seeking
16	Yadav et al. (58)	Cross-sectional	120,306	Self-reported illness including cancers, endocrine, metabolic and nutrition, cardiovascular and respiratory diseases	Uttar Pradesh	Community	Treatment-seeking
17	Nailwal et al. (43)	Cross-sectional	233	Non-communicable diseases	Uttarakhand	Facility	Treatment-seeking
18	Babu et al. (7)	Cross-sectional	7,590	Hypertension	Ladakh, Himachal Pradesh, Karnataka, Meghalaya, Odisha	Community	Treatment-seeking
19	Boro et al. (59)	Longitudinal	29,383	Hypertension	Pan Indian (Except Sikkim)	Community	Treatment-seeking
20	Chakraborty et al. (60)	Cross-sectional	300	Hypertension	Birbhum district of West Bengal	Community	Treatment-seeking
21	Chinnakali et al. (61)	Cross-sectional	211	Hypertension	Puducherry	Community	Treatment-seeking
22	Gupta et al. (62)	Longitudinal	100	Hypertension	Haryana	Facility	Treatment-seeking
23	Krishnamoorthy et al. (8)	Secondary data analysis	631,876	Hypertension	Pan Indian	Community	Treatment-seeking
24	Bhatia et al. (63)	Longitudinal	65,562	Hypertension	NI	Community	Treatment-seeking
25	Bharucha et al. (64)	Cross-sectional	2,879	Hypertension	Mumbai	Community	Treatment-seeking
26	Basu et al. (21)	Cross-sectional	788,974	Hypertension	New Delhi	Facility	Treatment-seeking
27	Balsari et al. (65)	Longitudinal	5,302	Hypertension	Nasik and Trimbakeshwar, Maharashtra	Community	Treatment-seeking
28	Sudharsanan et al. (66)	Cross-sectional	833	Hypertension	Chennai, Tamil Nadu	Community	Treatment-seeking
29	Singh et al. (67)	Cross-sectional	640	Hypertension	Varanasi, Uttar Pradesh	Community	Treatment-seeking
30	Singh et al. (68)	Cross-sectional	8,850	Hypertension	Northeast district of Delhi	Community	Treatment-seeking
31	Sharma et al. (10)	Cross-sectional	400	Multi-morbidity including diabetes, hypertension, COPD* and stroke	Himachal Pradesh	Community	Treatment-seeking
32	Rakesh et al. (69)	Randomized control trial	5,980	Hypertension	Kerala	Community	Treatment-seeking
33	Laxmaiah et al. (70)	Cross-sectional	47,401	Hypertension	Pan India	Community	Treatment-seeking
34	Sreekutty et al. (71)	Cross-sectional	333	Breast cancer	Thiruvananthapuram, Kerala	Facility	Delay in treatment-seeking
35	Kaku et al. (23)	Retrospective study	349	Cervical cancer	Thiruvananthapuram, Kerala	Facility	Delay in treatment-seeking
36	Krishnan et al. (27)	Cross-sectional	323	Cardiovascular diseases	Haryana	Community	Delay in treatment-seeking
37	Kumar et al. (24)	Cross-sectional	469	Breast cancer	Assam	Facility	Delay in treatment-seeking
38	Mohan et al. (28)	Prospective analytical study	619	Myocardial infarction	Punjab	Facility	Delay in treatment-seeking
39	Pakseresht et al. (25)	Cross-sectional	172	Breast cancer	New Delhi	Facility	Delay in treatment-seeking
40	Panda et al. (29)	Cross-sectional	130	Acute coronary syndrome	Chandigarh	Facility	Delay in treatment-seeking
41	Rai et al. (72)	Prospective analytical study	300	Breast cancer	Rishikesh	Facility	Delay in treatment-seeking
42	Somanna et al. (73)	Cross-sectional	210	Cervical cancer	Bengaluru	Facility	Delay in treatment-seeking
43	Somanna et al. (74)	Cross-sectional	392	Breast cancer	Bengaluru	Facility	Delay in treatment-seeking
44	Venkatesan et al. (30)	Cross-sectional	93	Acute myocardial infarction	Coimbatore	Facility	Delay in treatment-seeking
45	Agrawal et al. (26)	Cohort study	100	Acute coronary syndrome	Purvanchal	Facility	Delay in treatment-seeking
46	Zaman et al. (75)	Cross-sectional	11,657	Cardio metabolic diseases	Kerala and Andhra Pradesh	Facility	Preferred health facility
47	Verma et al. (17)	Cross-sectional	402	Metabolic syndrome	Rajasthan	Facility	Treatment-seeking
48	Arjun et al. (76)	Cross-sectional	740	COPD* or asthma	Trivandrum, Kerala	Facility	Preferred health facility and system of medicine
49	Baishya et al. (22)	NI	297	Head and neck cancer	North East India (Guwahati)	Facility	Delay in treatment-seeking
50	Gangane et al. (77)	NI	212	Breast cancer	Wardha, Maharashtra	Facility	Delay in treatment-seeking
51	Barbhuiya et al. (78)	Cross-sectional	100	Tobacco-related cancer	Barak Valley region of Assam	Facility	Initial treatment trajectory
52	Kishore et al. (79)	Cross-sectional	95	Cancer	New Delhi	Facility	Initial treatment trajectory
53	Kumar et al. (9)	Cross-sectional	192	Head and neck cancer	Puducherry	Facility	Initial treatment trajectory
54	Sarkar et al. (80)	Cross-sectional	113	Gynecological malignancies	West Bengal	Facility	Initial treatment trajectory
55	Vallabhajosyula et al. (81)	NI	303	Non-Hodgkin’s lymphoma	Manipal	Facility	Treatment-seeking
56	Raghuveer et al. (82)	Cohort	2,697	Diabetes	Mangaluru, Karnataka	Community	Screening uptake
57	Basu et al. (31)	Cross-sectional	469	Cervical cancer screening	Kolkata, West Bengal	Facility	Screening uptake
58	Khanna et al. (36)	Cross-sectional	290	Cervical cancer screening	Varanasi, Uttar Pradesh	Facility	Screening uptake
59	Nene et al. (32)	Randomized control trial	100,800	Cervical cancer screening	Four sub-districts of the Osmanabad district in Maharashtra	Facility	Screening uptake
60	Roy et al. (33)	Cross-sectional	299	Cervical cancer screening	Kolkata, West Bengal	Facility	Screening uptake
61	Sankaranarayanan et al. (34)	Randomized control trial	48,225	Cervical cancer screening	Dindigul District, Tamil Nadu	Community	Screening uptake
62	Ramagiri et al. (37)	Interventional study	267	Diabetic retinopathy screening among diabetics	Hyderabad	Community	Screening uptake
63	Gadgil et al. (35)	Interventional study	22,500	Breast cancer screening	Mumbai, Maharashtra	Community	Screening uptake
64	Singh et al. (38)	Interventional study	8,954	Diabetic retinopathy	Maharashtra	Facility	Screening uptake

*COPD, Chronic Obstructive Pulmonary Disorder.

### Data analysis

Data was extracted independently by two authors (HM & DN) in a data extraction form created in MS Excel 2013. From the included studies, general study information, design, sample size, location, population demographics, disease prevalence, treatment-seeking behavior, reasons for not seeking treatment, preferred health facility and system of medicine and factors associated with the health seeking behavior were extracted. Risk of bias assessment was conducted independently by two reviewers (RRL and SPJ) using Appraisal tool for Cross-Sectional Studies (AXIS) tool for all the included studies. The AXIS tool consists of 20 items that cover key domains such as the clarity of study aims, appropriateness of study design, justification of sample size, definition and selection of the target population, measurement validity and reliability, risk of non-response bias, description and appropriateness of statistical methods, ethical approval, and funding declarations, as well as clarity and transparency of results and conclusions. Each item is evaluated with one of the following responses: Yes, No, or Do not know, providing a structured and comprehensive framework for critical appraisal of the studies’ methodological quality (13).

Meta-analysis of proportions was performed using the DerSimonian and Laird random-effect model (14). The random-effects model was chosen because it assumes that the true effect sizes may vary between studies due to differences in study populations, settings, or methodologies. This model accounts for both within-study variance and between-study heterogeneity, making it more appropriate for combining results from observational studies. Visual assessment of the forest plots, the Cochran-Q test, and I-squared (*I*^2^) statistics were used to assess heterogeneity among the included studies. The *I*^2^ value greater than 25% or the Cochrane- Q less than 0.1 was considered to be the indicator of heterogeneity between the included studies. The source of heterogeneity was further investigated by the sub-group analysis. Publication bias was assessed using Funnel plot or Eggers test (15). Meta-analysis was performed using Stata version 18 SE (16). Two-sided *p* < 0.05 was considered to be statistically significant except for the heterogeneity test, wherein *p* < 0.10 was considered significant. Studies which did not provide sufficient data for meta-analysis were synthesized qualitatively/narratively.

## Results

**Figure 1:**
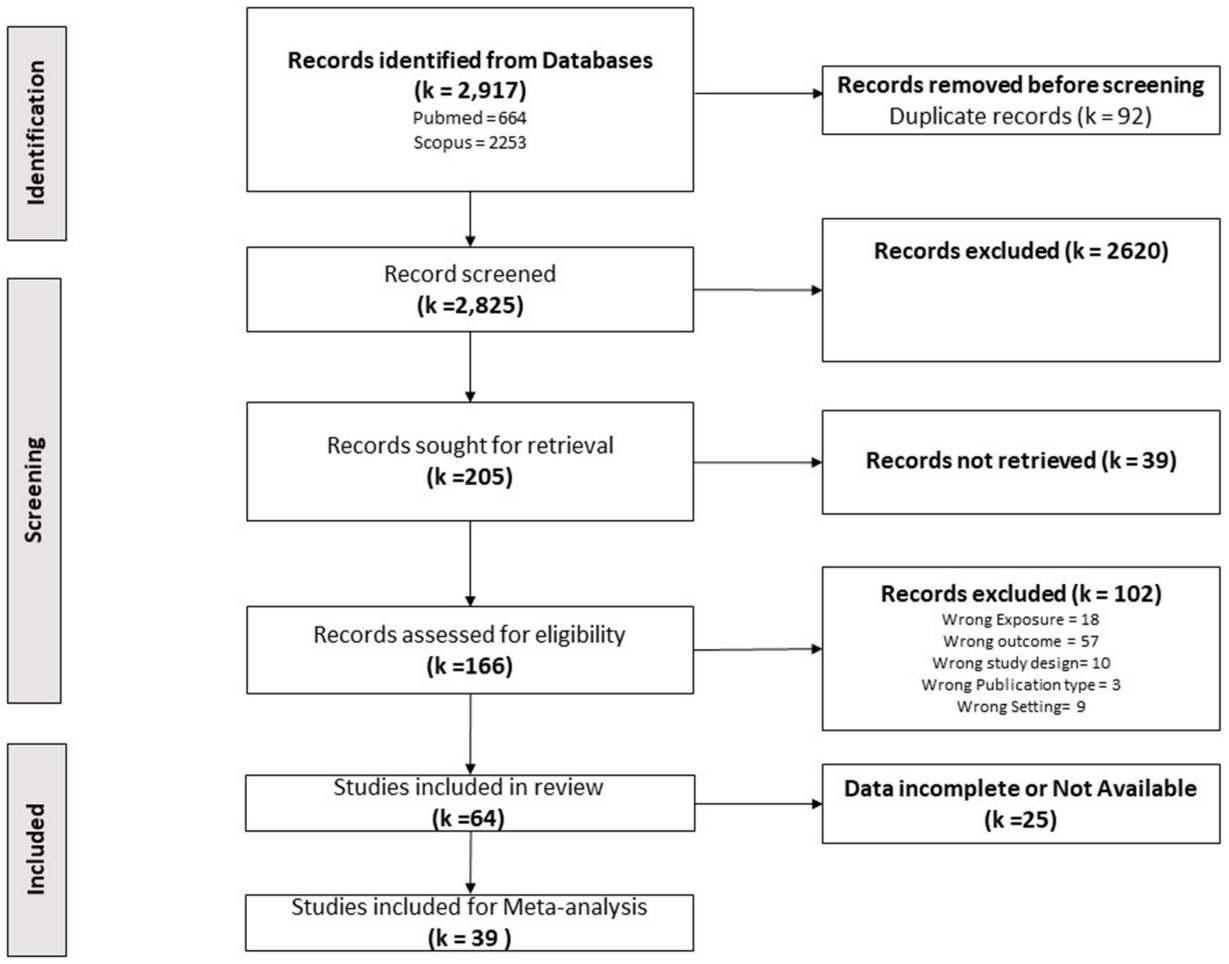
Study selection. PRISMA flow chart showing the total number studies identified in search, duplicates removed, studies excluded and included for the review.

A systematic search of PubMed and Scopus databases retrieved a total of 2,917 studies. After removing 92 duplicates, 2,825 studies were screened by title and abstract. From these, 205 studies were considered relevant for full-text retrieval. Despite efforts to contact corresponding authors, the full text of 39 studies could not be obtained. Out of the 166 studies assessed for full-text eligibility, 64 met the inclusion criteria and were included in the SRMA. The remaining 102 studies were excluded due to wrong exposure, outcome, study design, publication type, or setting, with list of reasons for exclusion and studies for exclusion provided in Supplementary Table S3. Of the 64 included studies, only 39 reported quantitative data suitable for meta-analysis. The results of the literature search, screening, and study selection process are documented in the PRISMA flowchart (Figure 1).

Among the 64 included studies for SRMA, 36 studies were focussed on treatment-seeking for major NCDs including hypertension (16 studies), diabetes (six studies), cardiovascular disease (two studies) and Chronic Obstructive Pulmonary Disease (one study). Eleven studies were conducted within the general population, investigating the prevalence of Non-Communicable Diseases (NCDs), notably diabetes and hypertension, along with the health-seeking behaviors associated with them. Further, 14 studies addressed either presentation/treatment delays in individuals with cancer and acute cardiac conditions. Presentation delay refers to the time taken to seek formal consultation after the appearance of first symptoms, while treatment delay pertains to the time taken to initiate the appropriate treatment after diagnosis. Lastly, eight studies examined the screening uptake/willingness to undergo screening as a health-seeking behavior.

### Qualitative synthesis

Eight out of the 36 studies on treatment-seeking for major NCDs, lacked quantitative data and hence, were included only for the systematic review. As per Verma et al. (17), 30% had good knowledge regarding CVD risk factors while only 9% engaged in implementing lifestyle measures. Inconsistent healthcare seeking behavior was reported among diabetic patients by Nimesh et al. (18) influenced by combination of factors including lack of improvement, affordability, accessibility, and healthcare personnel’s professional conduct. Shukla et al. (19) reported that the scale of city, type of the healthcare facility and education level had a statutory impact on health outcomes of patients afflicted with diabetic retinopathy.

Moreover, Srinivas et al. (20) reported that the noncompliance rate were extremely high among diabetic patients (57%), which was augmented by poor access to healthcare systems. Basu et al. (21) found out that treatment-seeking behaviors were absent among economically marginalized groups including rural dwellers and women. They reported 36.4% of individuals were hypertensive, while 48.5% were unaware about their condition (21).

Nine out of the 14 studies focussing on delay in treatment-seeking for cancer or acute cardiac conditions lacked sufficient quantitative data for meta-analysis and hence, were considered only for the systematic review. Health literacy and healthcare access were identified as potential strategies to mitigate delays and enhance health outcomes in four studies (22–25). Five studies focussed on presentation/treatment delays for acute cardiac conditions and stroke, highlighting the need for improving access to primary, secondary and tertiary health care facilities (26–30).

Eight studies described screening uptake out of which only six studies focussed on cancer and the remaining two focussed on diabetic retinopathy. None of the above mentioned studies provided consistent data to be included for meta-analysis, thereby limiting their inclusion only for systematic review. Studies focussing on Cancer screening revealed the influence of sociocultural barriers and demographic factors in screening uptake (31, 32). Additionally, low Pap test utilization was also reported among women which highlighted the need for effective cervical cancer screening strategies (33). Sankaranarayanan et al. (34) and Gadgil et al. (35) demonstrated the effectiveness of community-level screening strategies for cervical and breast cancer screening. Khanna et al. (36) stressed on the gap between awareness and actual participation in cervical cancer screening among community healthcare workers. Diabetic retinopathy screening studies emphasized that sustained use of health literacy videos as well as proximity to the healthcare facilities increased the screening uptake (37, 38).

### Quantitative synthesis (meta-analysis with subgroup analyses)

Among the total number of included studies, only 39 provided quantitative data, which were subjected to meta-analysis. These articles focused on the health seeking behaviors which included the proportion of individuals seeking treatment for an existing health condition, the preference for government versus private healthcare facilities, the choice between allopathy and alternative medicine, the proportion of individuals not seeking treatment for varied reasons, the proportion of individuals experiencing delays in seeking treatment and the reasons behind treatment delays were pooled in the meta-analysis.

#### Prevalence of individuals with NCDs seeking treatment

**Figure 2:**
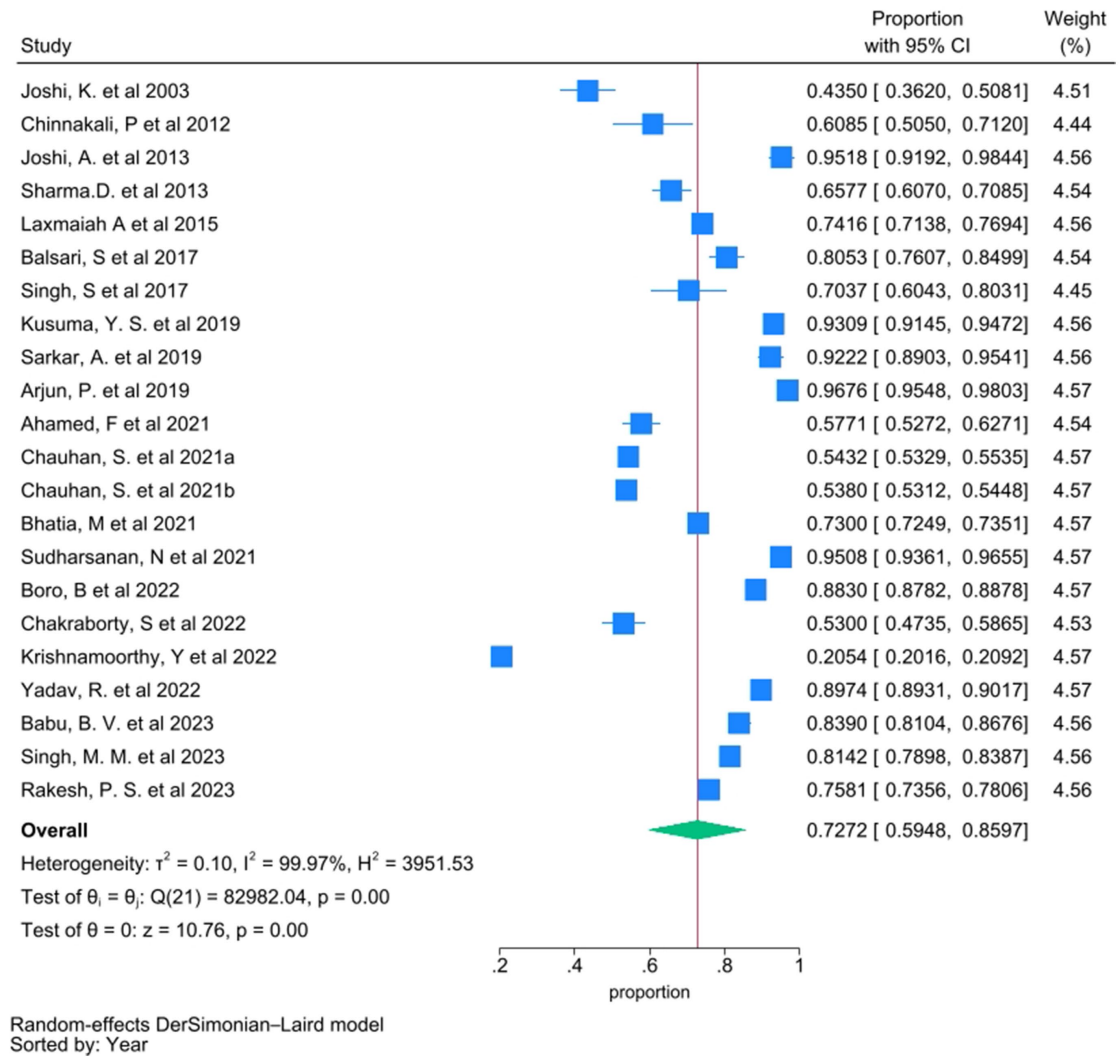
Forest plot showing pooled proportion of individuals who sought treatment for their existing disease condition. Proportion from individual studies was calculated by dividing the number of individuals who sought treatment with total number of individuals who had NCD/were aware of their disease condition, and synthesized by meta-analysis using random effect Dersimonian Laid model. Chauhan, S. et al. 2021a and Chauhan, S. et al. 2021b are same study with “a” indicating diabetic group and “b” indicating hypertensive group.

Among the studies included, data regarding seeking treatment for existing disease conditions were reported in 20 studies. The meta-analysis of the proportion of individuals seeking treatment revealed a pooled proportion of 0.7272 (95% CI 0.5948–0.8597), with a significant heterogeneity (*I*^2^ = 99.97%) (Figure 2). To investigate the source of this heterogeneity, subgroup analyses were conducted based on disease conditions, age of the study population, study location (urban/rural/tribal), and study setting (community/facility) (Supplementary Figures S1–S4).

#### Patterns of healthcare utilization

**Figure 3:**
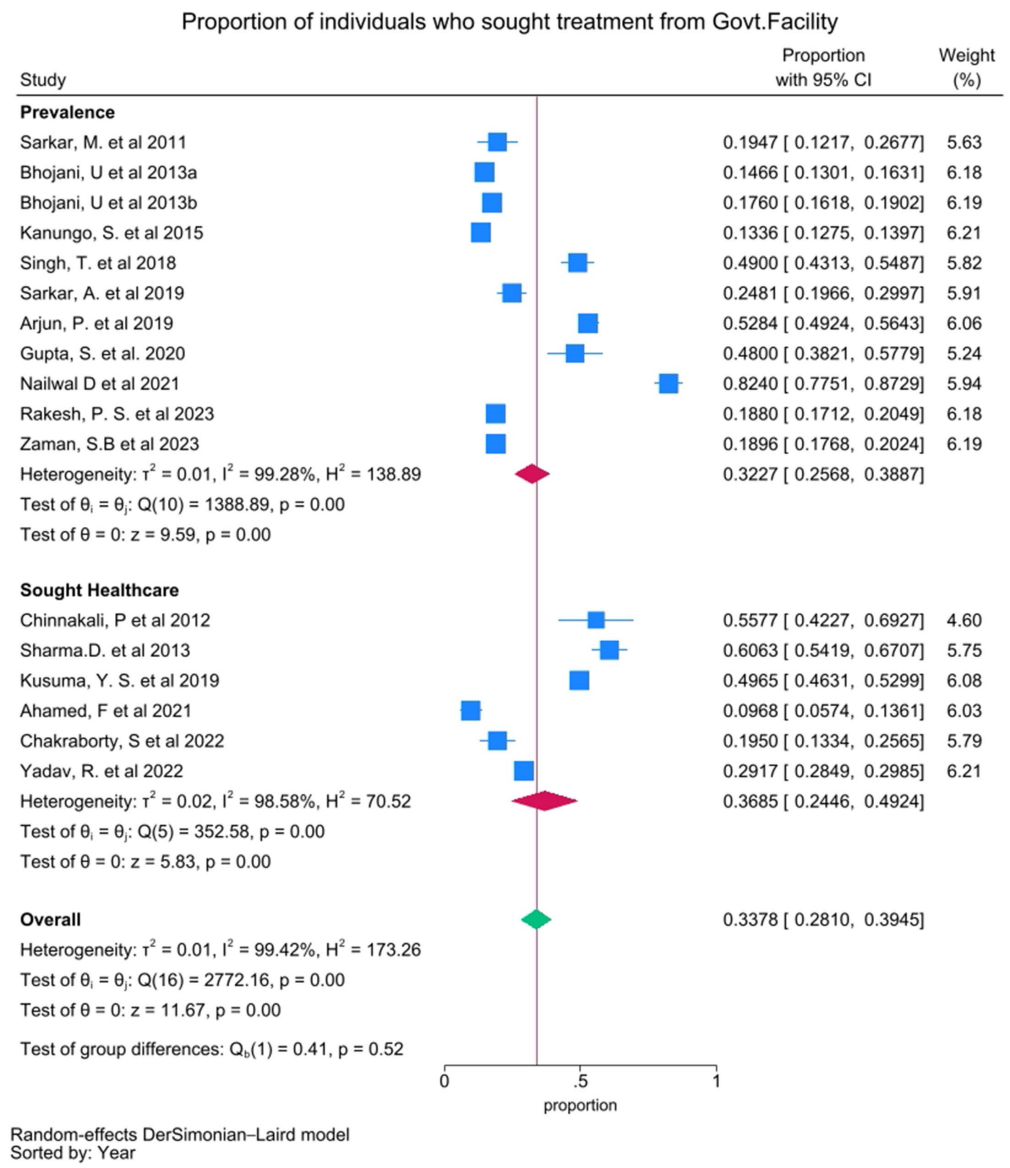
Forest plot showing pooled proportion of individuals who sought treatment from Government healthcare facilities. Proportion from individual studies was calculated by dividing the number of individuals who sought treatment from Government healthcare facilities with total number of individuals had NCD/sought treatment for NCD (presented as sub-groups), and synthesized by meta-analysis using random effect Dersimonian Laid model. Bhojani, U. et al. 2013a and Bhojani, U. et al. 2013b are same study with “a” indicating diabetic group and “b” indicating hypertensive group.

**Figure 4:**
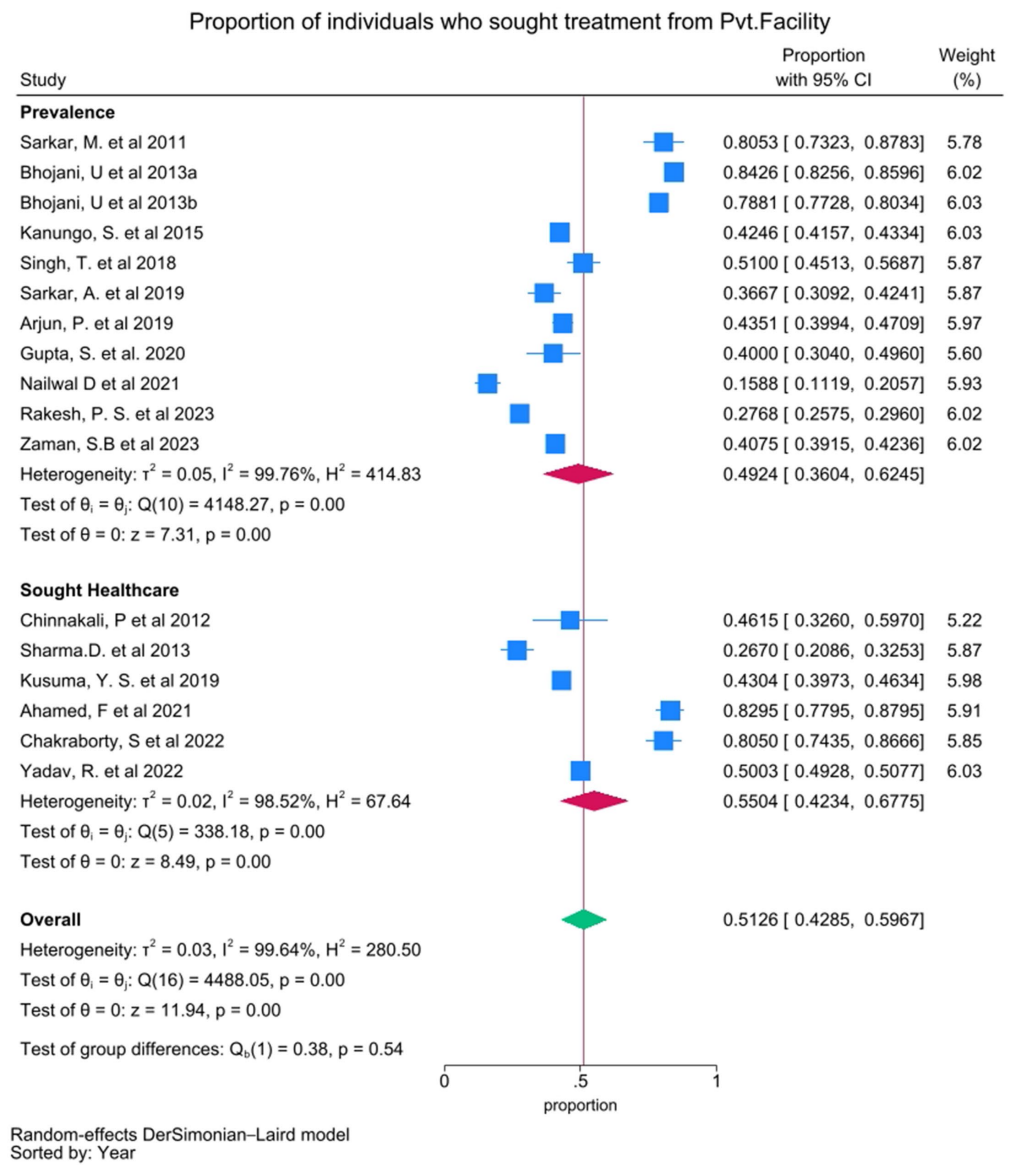
Forest plot showing pooled proportion of individuals who sought treatment from Private healthcare facilities. Proportion from individual studies was calculated by dividing the number of individuals who sought treatment from Private healthcare facilities with total number of individuals had NCD/sought treatment for NCD (presented as sub-groups), and synthesized by meta-analysis using random effect Dersimonian Laid model. Bhojani, U. et al. 2013a and Bhojani, U. et al. 2013b are same study with “a” indicating diabetic group and “b” indicating hypertensive group.

Twenty studies examined the preferred type of health facilities for treatment, including government/public health facilities, private healthcare facilities, and unqualified practitioners. These studies reported the proportion of individuals seeking treatment from public or private facilities, either among those with the disease (prevalence) or those who sought treatment, which are presented as sub-groups in the forest plot analysis. The pooled proportion of individuals seeking treatment from government health facilities was 0.3378 (95% CI 0.2810–0.3945), with substantial heterogeneity (*I*^2^ = 99.42%) (Figure 3). The pooled proportion for the individual seeking care from private health facilities was 0.5126 (95% CI 0.4285–0.5967), also with substantial heterogeneity (*I*^2^ = 99.64%) (Figure 4).

#### Preferred system of medicine

The choice between allopathic and alternative medicine was another frequently reported health-seeking behavior in the literature (10, 39–43). Our meta-analysis revealed that 73.09% (95% CI 54.01–92.16%, *I*^2^ = 99.18%) of individuals preferred allopathy, while 8.89% (95% CI 5.56 to 12.22%, *I*^2^ = 86.73%) opted for alternative medicine as their treatment choice for existing conditions, though there was substantial heterogeneity in the findings (Supplementary Figures S5, S6).

#### Reasons for not seeking treatment

Among the studies included for the meta-analysis, only a few studies stated the reasons for not seeking treatment. The most common reason for not seeking treatment was that the “illness was not considered to be serious.” The pooled proportion of individuals who cited this reason was 0.4785 (95% CI 0.4556–0.5013), based on data from four studies (Supplementary Figure S7). Financial constraints were the next major reason for not seeking treatment. The pooled proportion from four studies was 0.3263 (95% CI 0.1457–0.5069) (Supplementary Figure S8). Inaccessibility to healthcare facilities was another frequent reason, with a pooled proportion of 0.1329 (95% CI 0.0236–0.2422, *I*^2^ = 96.27%), based on five studies (Supplementary Figure S9). A smaller proportion of individuals reported lack of relief from treatment along with lack of trust in hospitals as the fundamental reason for not opting treatment (Supplementary Figures S10, S11). Other reasons included lack of family support, relying on faith for a cure, non-compliance with treatment, such as side effects of medications or improvement without treatment were not included in the meta-analysis due to insufficient data.

#### Reasons for delays in seeking treatment

Delays in seeking treatment were reported in 14 studies. Of these, four studies focused on acute cardiac conditions, while 10 studies examined various types of cancer, including breast cancer, cervical cancer and head and neck cancer. For studies focused on cancer, the average time taken between the onset of symptoms and the first hospital visit ranged from 11 to 180 days.

Some studies have reported data about the choice of initial treatment trajectory as a plausible cause of delay before reaching the actual cancer care providers (CCPs). A meta-analysis of this data revealed that while 54.62% (95% CI 38.4–70.84%) of patients consulted an allopathic physician only 21.22% (95% CI 7.38–35.05%) approached alternative medicine practitioners. Approximately 12.47% (95% CI 9.99–14.95%) visited primary health centers (PHCs) while a miniscule number of 7.28% (95% CI 2.76–11.8%) patients consulted unqualified treatment providers (including quacks and faith healers) before reaching the CCPs (Supplementary Figures S12–S15).

The most common reason for the presentation delay was a lack of disease awareness, as reported in five studies. The pooled proportion of individuals who delayed seeking treatment due to lack of knowledge about their disease was 0.5091 (95% CI 0.0294 to 0.9888), though with very high heterogeneity (*I*^2^ = 99.89%) (Supplementary Figure S16). The second major reason for delay was the absence of pain, particularly in the case of a lump, as reported in four studies. The pooled proportion was 0.4502 (95% CI 0.3312–0.5692), with substantial heterogeneity (*I*^2^ = 91.4%) (Supplementary Figure S17). Financial constraints were cited as a reason for delay in four studies, with a pooled proportion of 0.1472 (95% CI 0.0222–0.2721) (Supplementary Figure S18). Other reasons for delay included fear of diagnosis and prioritizing family responsibilities over personal health (Supplementary Figures S19, S20).

### Assessment of publication bias and risk of bias

The funnel plots showed symmetry and Egger’s test indicated *p* = 1, suggesting no publication bias among the studies (Supplementary Figures S21–S23). ROB assessment revealed low risk of bias for the questions on study aim, design, target population, sample frame, outcome measurement tools, non-responders, risk factor and outcome variable measurement, statistical significance, reproducibility of methods, description of basic data, analyses description in methods, justification of discussion and conclusion, ethical approval and informed consent. However, high risk of bias was observed in terms of sample size justification (56%), selection bias (17%), outcome variables measured (64%), basic data description (47%), concerns about non-response bias (33%), description of non-responders (38%), consistency in results (83%), limitation (81%), funding sources or conflicts of interest (56%) (Supplementary Figures S24–S28).

## Discussion

We conducted a SRMA to assess the health seeking behavior and its determinants in the Indian population who were afflicted with NCDs covered under the NPCDCS program. Specifically, we focused on attributes including treatment-seeking behavior, screening uptake as well as the presentation and treatment delay.

Our meta-analysis of 22 studies revealed that 72.72% (Figure 1) of individuals in India sought treatment for existing health conditions, which is significantly higher than the global average of 56% (95% CI: 44–68) (44). Even within the global estimate, the highest treatment-seeking rate was reported in an Indian study to be 91% (45). Thus higher treatment seeking rate in India could be attributed to several factors, including increasing awareness of NCDs, improved accessibility of healthcare services and expanding coverage through schemes like Ayushman Bharat (46). However, we also observed significant heterogeneity in our findings. Sub-group analysis showed variations in treatment-seeking patterns based on disease condition, age, study locations and settings. The majority of the included studies focused on hypertensive patients, with a treatment-seeking rate of 71.87%. A single study on COPD reported the highest treatment-seeking rate at 96.76%, while a study on diabetes showed a lower rate of 54.32%, possibly due to perceived severity (Supplementary Figure S1). This also highlights a dearth of studies on COPD and diabetes. Studies covering all ages reported the highest treatment-seeking rate at 93.17%, while a single study analyzing National Family Health Survey-4 data involving young adults with hypertension (<45 years) reported the lowest rate at 20.54% highlighting the need for age-specific strategies (Supplementary Figure S2).

Sub-groups based on studies conducted on populace residing in urban and rural areas, revealed higher treatment-seeking behavior in urban areas (84.82%) as compared to rural areas (68.04%) (Supplementary Figure S3) highlighting the disparities in healthcare access and utilization between these settings. According to Banerjee (47), education and the economic status were observed to be the major factors for the variances in the disease prevalence between the urban and rural milieus. Thus to overcome these disparities the demand is to develop nuanced niche specific surveillance strategies to promote health literacy and improve health outcomes in rural regions of the Indian sub-continent.

In contrast to the rural settings, health seeking behavior for hypertension among tribal population was observed to be higher (79.02%) (Supplementary Figure S3) among the individuals who are aware of their disease condition. However, disease awareness seems to be very poor in this population. Therefore, screening for hypertension in tribal populations is crucial to alleviate the burden of hypertension associated complications in these marginalized and under-served populations.

Facility-level studies showed higher treatment-seeking rates (96.55%) compared to community based studies (70.39%) (Supplementary Figure S4) whose discrepancy could be attributed to a selection bias, as individuals visiting a facility were more likely to be on treatment for their disease conditions. Therefore, community based studies maybe more reliable for assessing the health seeking behavior since they use a more representative sample of the population.

Our analyses revealed that private healthcare facilities were preferred in comparison to the government healthcare facilities (51.26% vs. 33.78%) (Figure 3). This disparity in preference of healthcare facilities underscores the need for community empowering ‘bottoms-up’ policies along with the strengthening of the public health infrastructure to alleviate the extant disparities with respect to accessibility and quality. Our results indicated a higher preference for the allopathic system of medicine (73.09%) compared to the alternative systems (8.89%) (Supplementary Figures S5, S6), based on the available studies. The reported results might have been influenced as most of the included studies were conducted in facilities delivering allopathic treatment. Additional investigations regarding AYUSH utilization in NCD management become essential to understand its role in India’s diverse healthcare environment. “Illness not considered to be serious” was the predominant reported reason (47.85%) for not seeking healthcare which was followed by “financial constraints” (32.63%), “non-accessibility” to healthcare facilities (13.29%), “no cure” perception (5.92%), and “distrust in hospitals” (2.95%) (Supplementary Figures S7–S11) which necessitates promotion of affordable and accessible healthcare facilities across 806 districts (and counting) of the Indian sub-continent to improve the health outcomes.

Regarding cancer as well as acute cardiac conditions, 14 studies reported presentation/treatment delay along with “Lack of awareness” (50.91%) as the major factor contributing to poor health outcomes. This forms the rationale for enhancing health literacy among the populace regarding the risk factors of various health conditions. Other factors cited were “painlessness” of cancer lumps (45.02%) followed by “fear of diagnosis” (5.84%) and “family priorities” (5.81%). Taken together, these factors indicate the confluence of misconceptions, psychosocial, and cultural barriers as taboos which hinder an individual from seeking the appropriate treatment options. Pro-active community engagement activities enmeshed with health literacy and health support campaigns are expected to significantly alleviate such barriers thereby improving timely access to affordable healthcare options.

Our SRMA highlighted certain evidence gaps toward understanding of health-seeking behavior for NCDs in the Indian sub-continent. There is a lack of broad representation of data at the pan-Indian level with majority of studies being restricted to geographically delimited areas. Additionally, current literature primarily studies healthcare-seeking behavior toward government and private providers yet overlooks the influence of unlicensed practitioners. In many rural regions without adequate healthcare facilities, people continue to seek treatment from unqualified practitioners. This should be one focus area of future research in order to comprehend the influence of such practices on health-seeking behavior. Structuring of inclusive community empowering ‘bottoms-up’ policies, along with automated allocation of the clinical resources is essential in a populace endowed with wide variation in genetic base inhabiting landscapes with unique geological relief structures contributing to segregated socio-cultural norms and practices. Furthermore, implementation of nuanced multifaceted approaches, such as Artificial Intelligence (AI)-enabled large-scale surveillance systems for now-casting and forecasting primary data, use of iterative and integrated dashboards with heuristic capabilities would be pivotal to address the diverse healthcare needs of the Indian populace (48). Establishment of Community Advisory Boards (CABs) in low- and middle-income countries, including India (49) are expected to be culturally sensitive and context specific promoting comprehensive health equality among the general populace. This in turn would construe as the platform toward the alignment with the United Nations charter of Sustainable Development Goals (SDGs).

Our study has several strengths. To the best of our knowledge, this is a pioneering effort in assessing the health-seeking behavior among the Indian population afflicted with NCDs across unique geological relief structures contributing to segregated socio-cultural norms and practices. We included a wide spectrum of NCDs to understand the intricate complexities of the health-seeking behavior comprehensively. Our study also has certain limitations. The inclusion of only quantitative studies depicting the patterns and processes associated with the health-seeking behavior of the Indian population might limit the scope of our understanding of the subtle variances in the health seeking behavior. Future research incorporating qualitative methodologies is needed to prefer a more comprehensive understanding of health-seeking behavior among the Indian populace.

## Data Availability

The original contributions presented in the study are included in the article/Supplementary material, further inquiries can be directed to the corresponding author.
