# Supplementary material for: Health-seeking behavior and its determinants for non-communicable diseases in India - a systematic review and meta-analysis
Source: Front Public Health. 2025 Jun 11;13:1580824. doi: 10.3389/fpubh.2025.1580824 (PMC12187832; doi:10.3389/fpubh.2025.1580824)
Supplement: Supplementary file 1 [file Data_Sheet_1.docx]

**Title: Health-seeking behaviour for Non-communicable diseases and its determinants in India- A Systematic Review and Meta-analysis**

**Authors:** Madhumitha Haridoss PhD^#^, Dhruva Nandi MPH, Raji Rajesh Lenin PhD, Shiny PJ PhD, Rajiv Janardhanan PhD^#^

**Supplementary Material**

**Supplementary Tables 1 & 2**

**Supplementary Figures 1-28**

**Supplementary Table 1. Search strategy and results in Pubmed**

| **Search #** | **Search Terms** | **Search hits as on 27-10-2023** |
| --- | --- | --- |
| **#1** | "Residence Characteristics"[Mesh] OR Community OR communit* OR village OR town OR tribal OR tribe OR neighbourhood OR neighborhood OR residents OR rural OR urban OR family OR families OR city OR cities | [**4,152,936**](https://pubmed.ncbi.nlm.nih.gov/?term=%22Residence+Characteristics%22%5bMesh%5d+OR+Community+OR+communit*+OR+village+OR+town+OR+tribal+OR+tribe+OR+neighbourhood+OR+neighborhood+OR+residents+OR+rural+OR+urban+OR+family+OR+families+OR+city+OR+cities&sort=date&ac=no) |
| **#2** | "Patient Acceptance of Health Care"[Mesh] OR Health-seeking OR help-seeking OR (health AND seek) OR (help AND seek) OR (health AND seeking) OR (help AND seek) OR (help AND seeking) OR treatment-seeking OR "treatment seeking" OR care-seeking OR “care seeking” OR "Health care utilization" | [**287,308**](https://pubmed.ncbi.nlm.nih.gov/?term=%22Patient+Acceptance+of+Health+Care%22%5bMesh%5d+OR+Health-seeking+OR+help-seeking+OR+(health+AND+seek)+OR+(help+AND+seek)+OR+(health+AND+seeking)+OR+(help+AND+seek)+OR+(help+AND+seeking)+OR+treatment-seeking+OR+%22treatment+seeking%22+OR+care-seeking+OR+%22care+seeking%E2%80%9C+OR+%22Health+care+utilization%22&sort=date&ac=no) |
| **#3** | "Noncommunicable Diseases"[Mesh] OR Non-communicable diseases OR NCD OR "Cardiovascular Diseases"[Mesh] OR cardio* OR "Cerebrovascular Disorders"[Mesh] OR cerebro* OR "Heart"[Mesh] OR heart OR "Stroke"[Mesh] OR stroke OR "Diabetes Mellitus, Type 2"[Mesh] OR diabetic OR diabetes OR "Obesity"[Mesh] OR obesity OR metabol* OR (insulin AND (resistance OR resistant)) OR dyslipid* OR "Hypertension"[Mesh] OR hypertension OR "Hypotension"[Mesh] OR hypotension OR "blood pressure“ OR "Non-alcoholic Fatty Liver Disease"[Mesh] OR “Metabolic Associated Fatty Liver Disease” OR NAFLD OR MAFLD OR “Fatty liver” OR "Neoplasms"[Mesh] OR cancer OR tumor OR carcino* OR malignan* OR “chronic respiratory” OR COPD OR “Chronic Obstructive Pulmonary Disease” OR “Chronic Kidney Disease” OR CKD | [**15,671,886**](https://pubmed.ncbi.nlm.nih.gov/?term=%22Noncommunicable+Diseases%22%5bMesh%5d+OR+Non-communicable+diseases+OR+NCD+OR+%22Cardiovascular+Diseases%22%5bMesh%5d+OR+cardio*+OR+%22Cerebrovascular+Disorders%22%5bMesh%5d+OR+cerebro*+OR+%22Heart%22%5bMesh%5d+OR+heart+OR+%22Stroke%22%5bMesh%5d+OR+stroke+OR+%22Diabetes+Mellitus,+Type+2%22%5bMesh%5d+OR+diabetic+OR+diabetes+OR+%22Obesity%22%5bMesh%5d+OR+obesity+OR+metabol*+OR+(insulin+AND+(resistance+OR+resistant))+OR+dyslipid*+OR+%22Hypertension%22%5bMesh%5d+OR+hypertension+OR+%22Hypotension%22%5bMesh%5d+OR+hypotension+OR+%22blood+pressure%E2%80%9C+OR+%22Non-alcoholic+Fatty+Liver+Disease%22%5bMesh%5d+OR+%E2%80%9CMetabolic+Associated+Fatty+Liver+Disease%E2%80%9D+OR+NAFLD+OR+MAFLD+OR+%E2%80%9CFatty+liver%E2%80%9D+OR+%22Neoplasms%22%5bMesh%5d+OR+cancer+OR+tumor+OR+carcino*+OR+malignan*+OR+%E2%80%9Cchronic+respiratory%E2%80%9D+OR+COPD+OR+%E2%80%9CChronic+Obstructive+Pulmonary+Disease%E2%80%9D+OR+%E2%80%9CChronic+Kidney+Disease%E2%80%9D+OR+CKD&sort=date&ac=no) |
| **#4** | India OR "India"[Mesh] | [**793,602**](https://pubmed.ncbi.nlm.nih.gov/?term=India+OR+%22India%22%5bMesh%5d&sort=date&ac=no) |
|  | #1 AND #2 AND #3 AND #4 | [**664**](https://pubmed.ncbi.nlm.nih.gov/?term=) |

**Supplementary Table 2. Search strategy and results in Scopus**

| **Search #** | Search Terms | **Search hits as on 27-10-2023** |
| --- | --- | --- |
| **#1** | community OR communit* OR village OR town OR tribal OR tribe OR neighbourhood OR neighborhood OR residents OR rural OR urban OR family OR families OR city OR cities | [**19,215,969**](https://www.scopus.com/search/history/results.uri?origin=searchhistory&shid=2) |
| **#2** | health-seeking OR help-seeking OR ( health AND seek ) OR ( help AND seek ) OR ( health AND seeking ) OR ( help AND seek ) OR ( help AND seeking ) OR treatment-seeking OR "treatment seeking" OR care-seeking OR "care seeking” OR "Health care utilization" | [**771,424**](https://www.scopus.com/search/history/results.uri?origin=searchhistory&shid=3) |
| **#3** | Non-communicable diseases OR NCD OR cardio* OR cerebro* OR heart OR stroke OR diabetic OR diabetes OR obesity OR metabol* OR (insulin AND (resistance OR resistant)) OR dyslipid* OR hypertension OR hypotension OR “blood pressure” OR “Metabolic Associated Fatty Liver Disease” OR NAFLD OR MAFLD OR “Fatty liver” OR cancer OR tumor OR carcino* OR malignan* OR “chronic respiratory” OR COPD OR “Chronic Obstructive Pulmonary Disease” OR “Chronic Kidney Disease” OR CKD | [**89,030**](https://www.scopus.com/search/history/results.uri?origin=searchhistory&shid=4) |
| **#4** | India | [**4,834,170**](https://www.scopus.com/search/history/results.uri?origin=searchhistory&shid=5) |
|  | #1 AND #2 AND #3 AND #4 | [**2,253**](https://www.scopus.com/search/history/results.uri?origin=searchhistory&shid=6) |

| **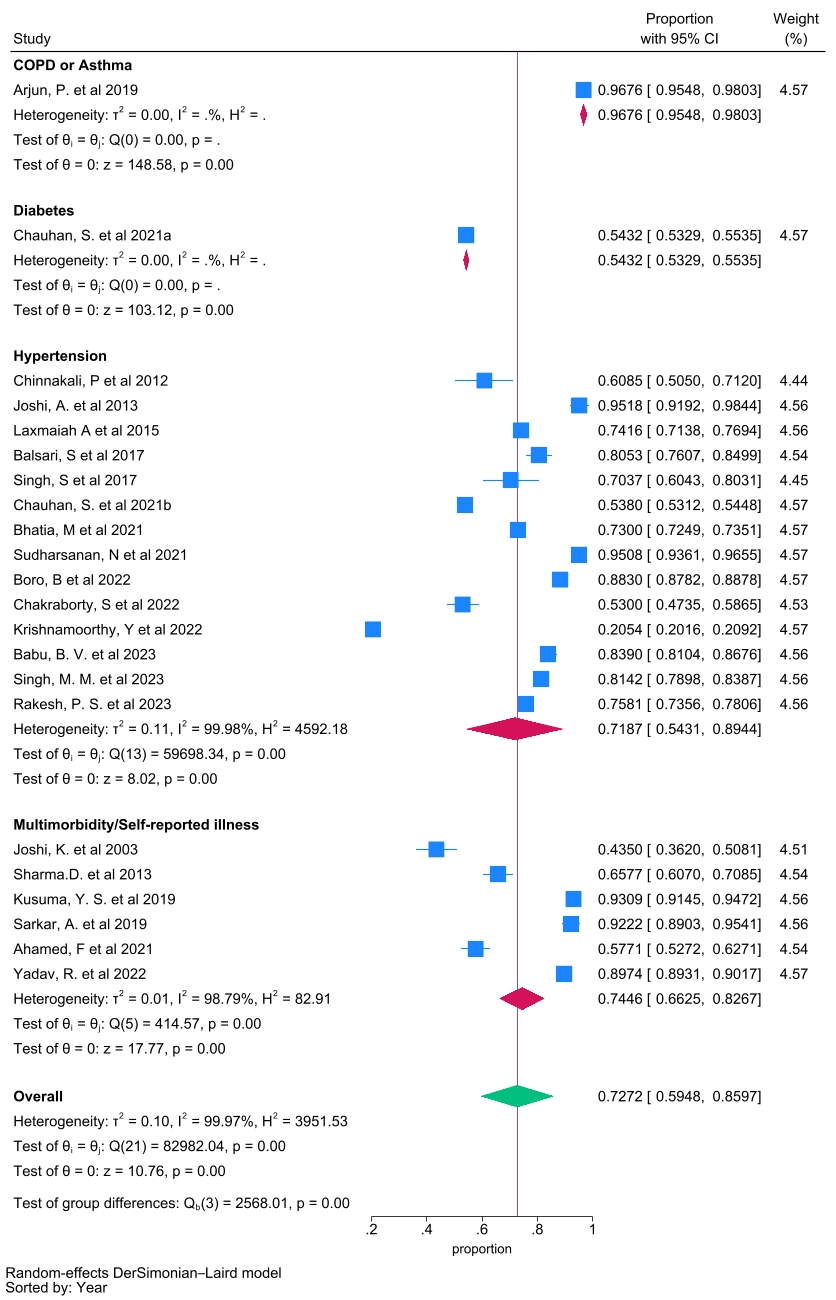** |
| --- |
| **Supplementary Figure 1. Forest plot showing pooled proportion of individuals who sought treatment and sub-grouped based on their existing disease condition** |

Proportion from individual studies was calculated by dividing the number of individuals who sought treatment with total number of individuals who had NCD/were aware of their disease condition, and synthesized by meta-analysis using random effect Dersimonian Laid model (Figure 1). Studies were then sub-grouped based on the disease condition. Studies on multimorbidity/self-reported illness include individuals with one or more health issues, with hypertension or diabetes being the most prevalent. Chauhan, S.et al 2021a and Chauhan, S.et al 2021b are same study with ‘a’ indicating diabetic group and ‘b’ indicating hypertensive group

| **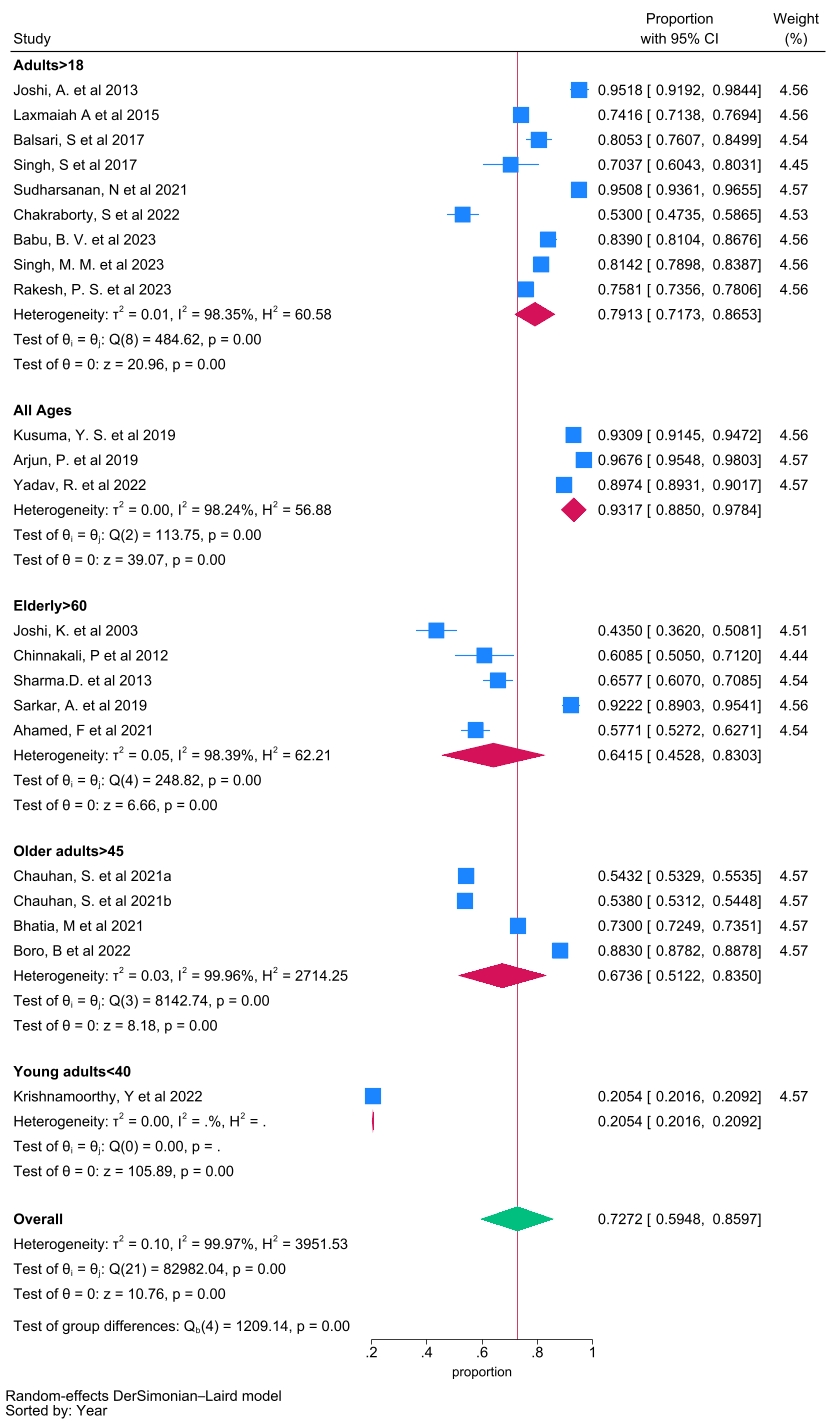** |
| --- |
| **Supplementary Figure 2. Forest plot showing pooled proportion of individuals who sought treatment and sub-grouped based on the age of the study population** |

Proportion from individual studies was calculated by dividing the number of individuals who sought treatment with total number of individuals who had NCD/were aware of their disease condition, and synthesized by meta-analysis using random effect Dersimonian Laid model (Figure 1). Studies were then sub-grouped based on the age of the study population. Chauhan, S.et al 2021a and Chauhan, S.et al 2021b are same study with ‘a’ indicating diabetic group and ‘b’ indicating hypertensive group

| **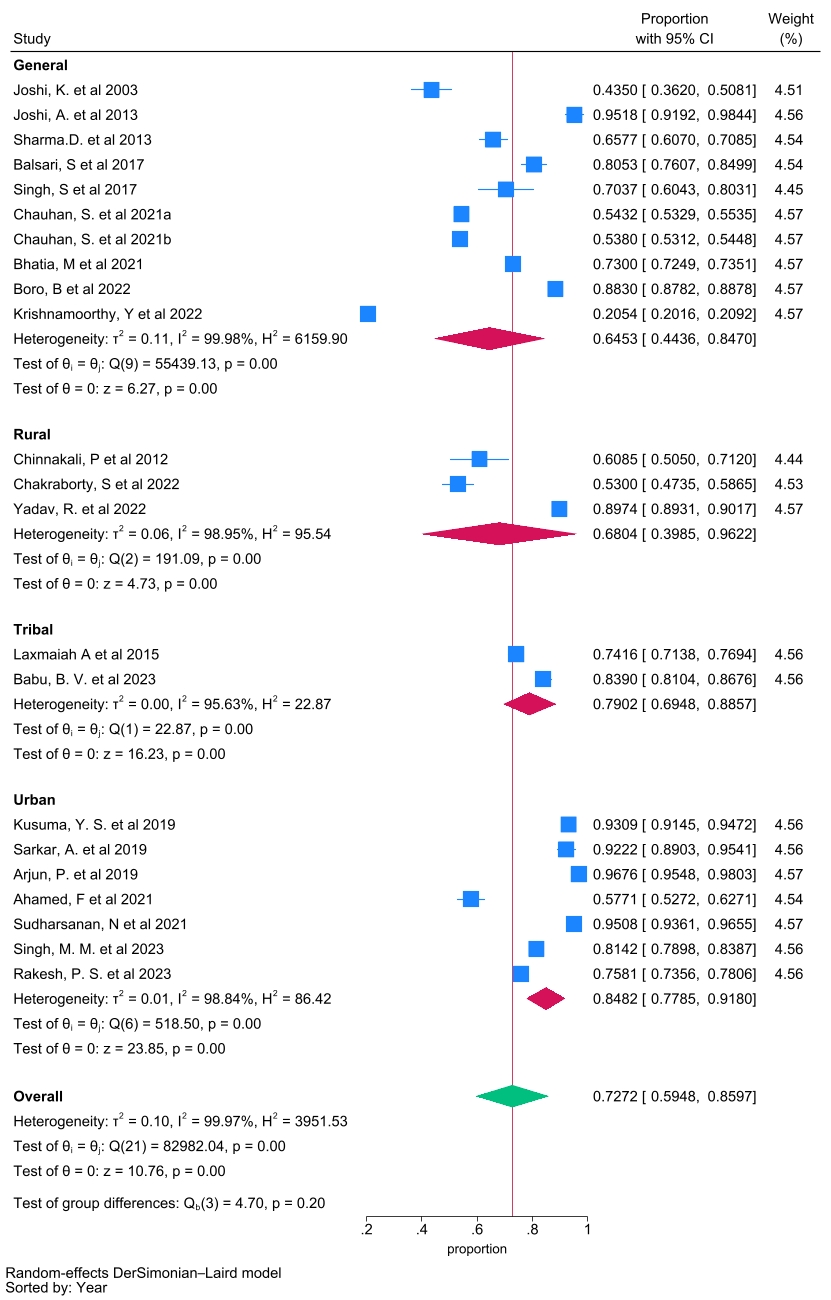** |
| --- |
| **Supplementary Figure 3. Forest plot showing pooled proportion of individuals who sought treatment and sub-grouped based on the study location**  Proportion from individual studies was calculated by dividing the number of individuals who sought treatment with total number of individuals who had NCD/were aware of their disease condition, and synthesized by meta-analysis using random effect Dersimonian Laid model (Figure 1). Studies were then sub-grouped based on different study locations including urban, rural and tribal. Sub-group general include both urban and rural population. Chauhan, S.et al 2021a and Chauhan, S.et al 2021b are same study with ‘a’ indicating diabetic group and ‘b’ indicating hypertensive group |
| **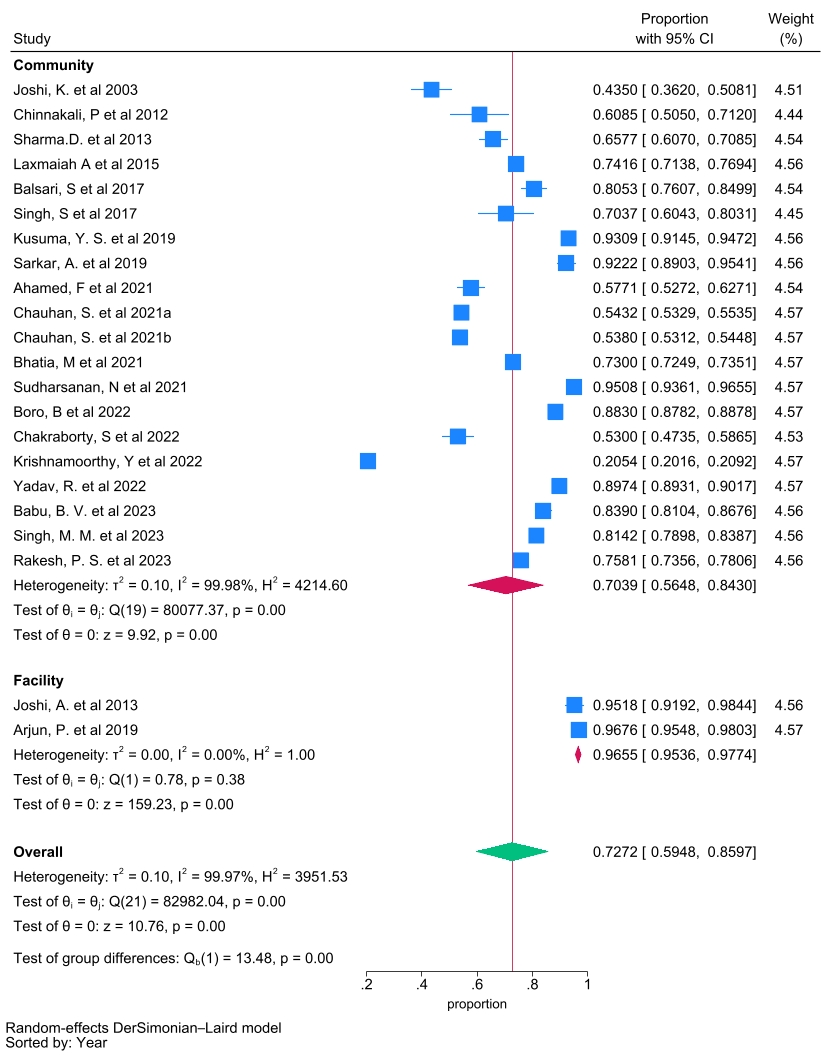** |
| **Supplementary Figure 4. Forest plot showing pooled proportion of individuals who sought treatment and sub-grouped based on the study setting** |

Proportion from individual studies was calculated by dividing the number of individuals who sought treatment with total number of individuals who had NCD/were aware of their disease condition, and synthesized by meta-analysis using random effect Dersimonian Laid model (Figure 1). Studies were then sub-grouped based on different study settings including urban, rural and tribal. Sub-group general include both urban and rural population. Chauhan, S.et al 2021a and Chauhan, S.et al 2021b are same study with ‘a’ indicating diabetic group and ‘b’ indicating hypertensive group

| **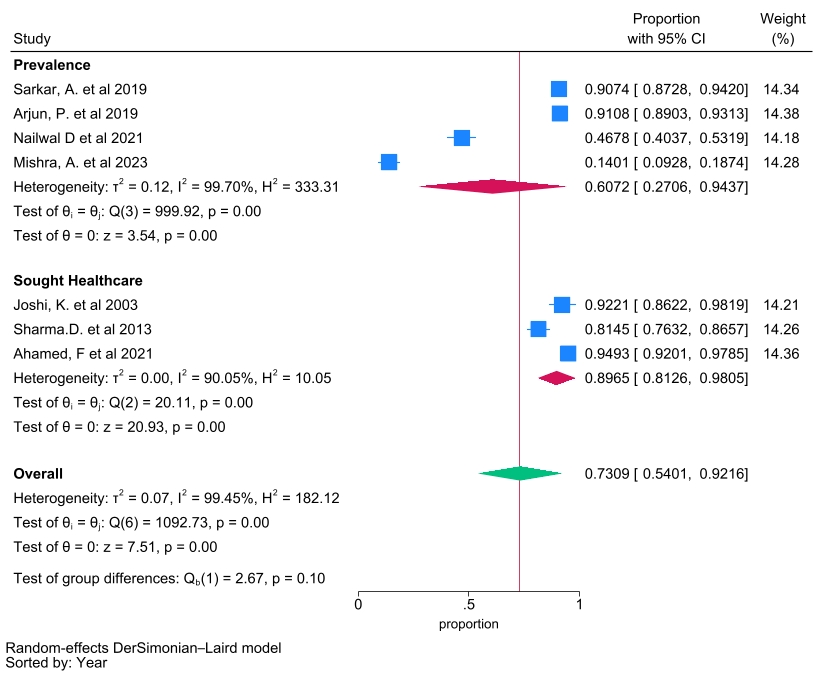** |
| --- |
| **Supplementary Figure 5. Forest plot showing pooled proportion of individuals who sought treatment using Allopathic system of medicine** |

Proportion from individual studies was calculated by dividing the number of individuals who sought treatment using Allopathic system of medicine with total number of individuals who had disease/sought treatment (presented as subgroups), and synthesized by meta-analysis using random effect Dersimonian Laid model

| **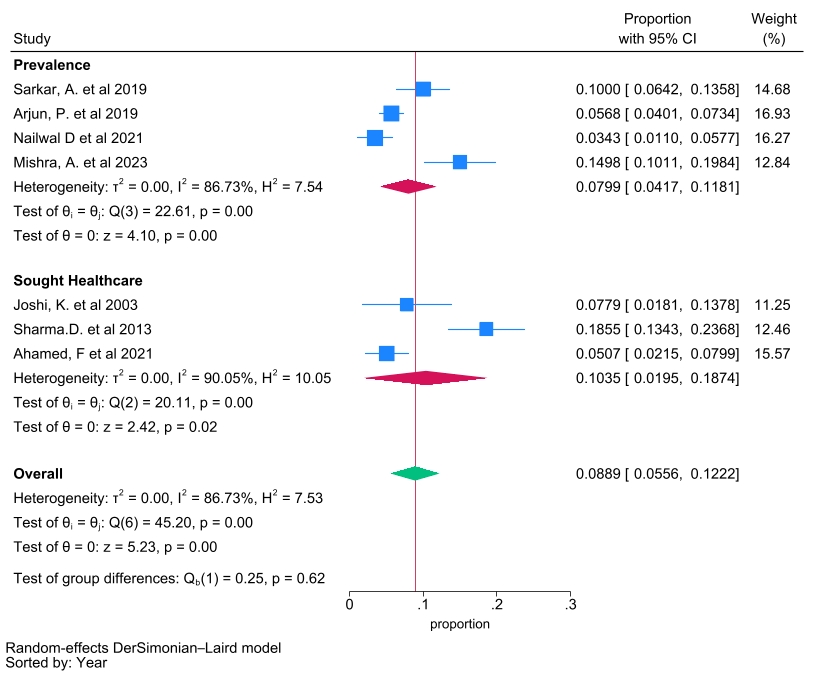** |
| --- |
| **Supplementary Figure 6. Forest plot showing pooled proportion of individuals who sought treatment using Alternative system of medicine** |

Proportion from individual studies was calculated by dividing the number of individuals who sought treatment using Alternative system of medicine with total number of individuals who had disease/sought treatment (presented as subgroups), and synthesized by meta-analysis using random effect Dersimonian Laid model

| **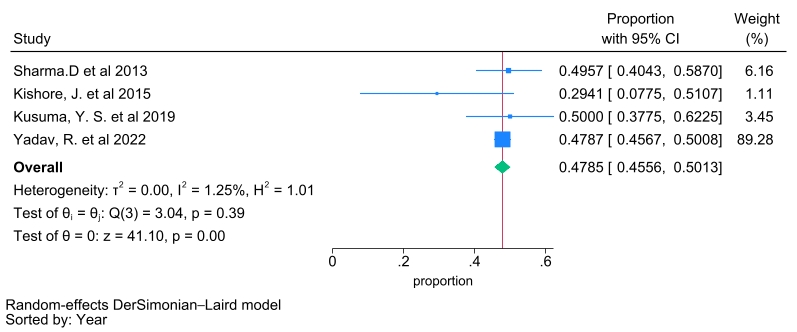** |
| --- |
| **Supplementary Figure 7. Forest plot showing pooled proportion of individuals who considered ‘illness as not serious’ as a reason for not seeking healthcare**  Proportion from individual studies was calculated by dividing the number of individuals who considered ‘illness as not serious’ as a reason for not seeking healthcare with total number of individuals who did not seek treatment, and synthesized by meta-analysis using random effect Dersimonian Laid model |
| **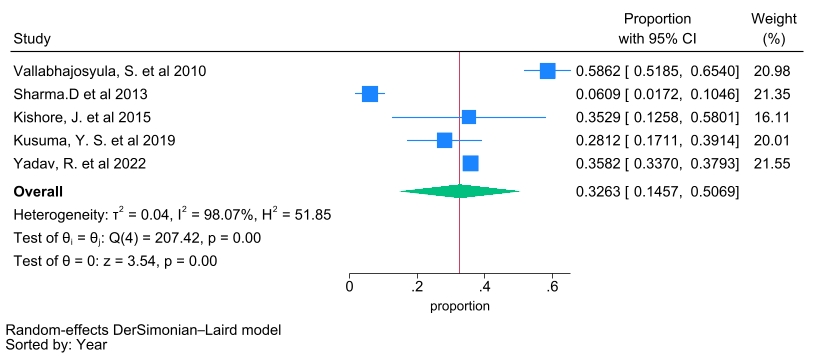** |
| **Supplementary Figure 8. Forest plot showing pooled proportion of individuals who considered ‘financial constraints’ as a reason for not seeking healthcare**  Proportion from individual studies was calculated by dividing the number of individuals who considered ‘financial constraints’ as a reason for not seeking healthcare with total number of individuals who did not seek treatment, and synthesized by meta-analysis using random effect Dersimonian Laid model |
| **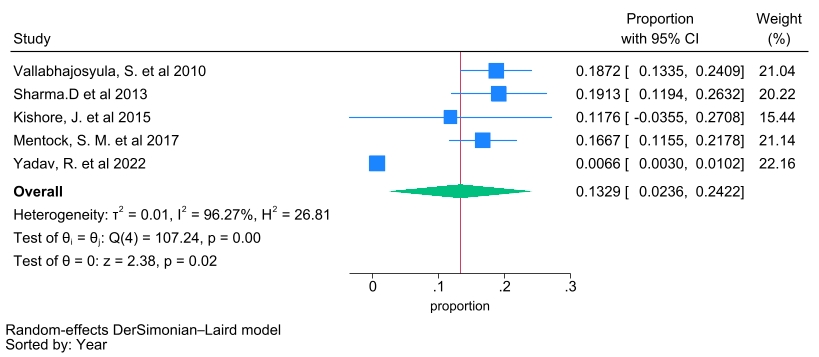** |
| **Supplementary Figure 9. Forest plot showing pooled proportion of individuals who considered ‘no accessibility’ as a reason for not seeking healthcare**  Proportion from individual studies was calculated by dividing the number of individuals who considered ‘no accessibility’ as a reason for not seeking healthcare with total number of individuals who did not seek treatment, and synthesized by meta-analysis using random effect Dersimonian Laid model |
| **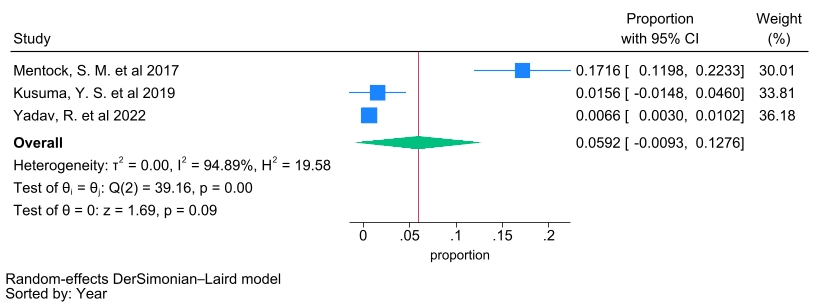** |
|  |
| **Supplementary Figure 10. Forest plot showing pooled proportion of individuals who considered ‘no cure for their disease’ as a reason for not seeking healthcare** |

Proportion from individual studies was calculated by dividing the number of individuals who considered ‘no cure for their disease’ as a reason for not seeking healthcare with total number of individuals who did not seek treatment, and synthesized by meta-analysis using random effect Dersimonian Laid model

| **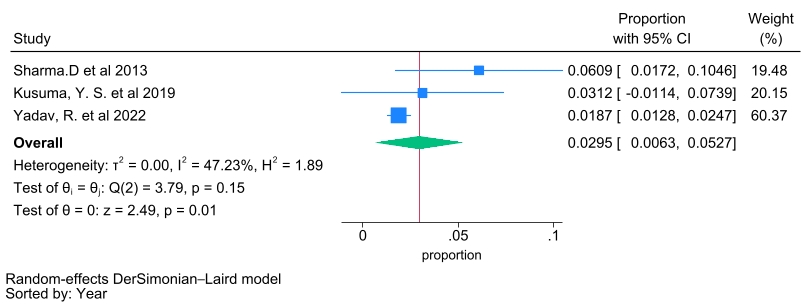** |
| --- |
| **Supplementary Figure 11. Forest plot showing pooled proportion of individuals who considered ‘distrust in healthcare settings’ as a reason for not seeking healthcare** |

Proportion from individual studies was calculated by dividing the number of individuals who considered ‘distrust in healthcare settings’ as a reason for not seeking healthcare with total number of individuals who did not seek treatment, and synthesized by meta-analysis using random effect Dersimonian Laid model

| **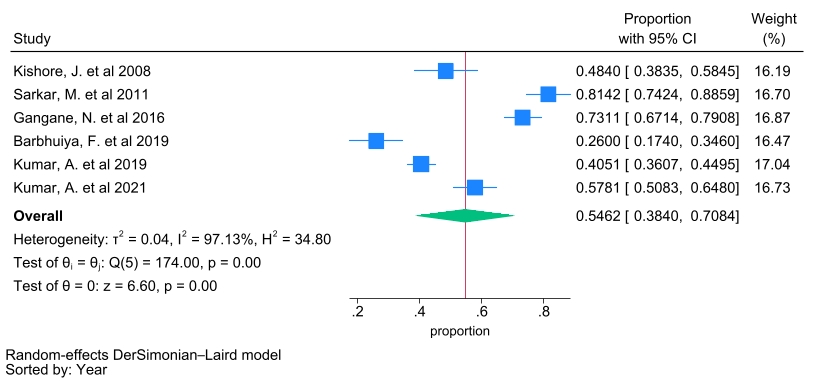** |
| --- |
| **Supplementary Figure 12. Forest plot showing pooled proportion of individuals who initially consulted Allopathic physician before reaching CCP** |

Proportion from individual studies was calculated by dividing the number of individuals who initially consulted Allopathic physician for their cancer symptoms with total study population and synthesized by meta-analysis using random effect Dersimonian Laid model. (CCP- Cancer care provider**)**

| **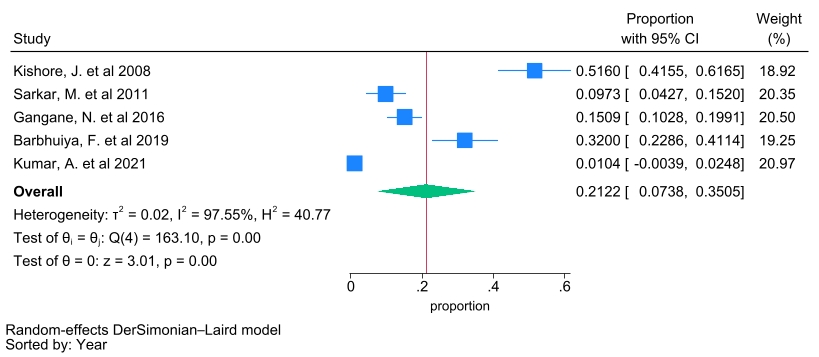** |
| --- |
| **Supplementary Figure 13. Forest plot showing pooled proportion of individuals who initially consulted Alternative medicine practitioner before reaching CCP**  Proportion from individual studies was calculated by dividing the number of individuals who initially consulted Alternative medicine practitioner for their cancer symptoms with total study population and synthesized by meta-analysis using random effect Dersimonian Laid model. (CCP- Cancer care provider**)** |

| **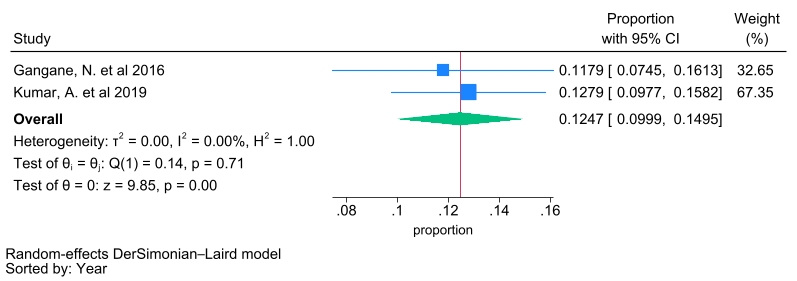** |
| --- |
| **Supplementary Figure 14. Forest plot showing pooled proportion of individuals who initially visited PHC before reaching CCP** |

Proportion from individual studies was calculated by dividing the number of individuals who initially visited PHC for their cancer symptoms with total study population and synthesized by meta-analysis using random effect Dersimonian Laid model. (CCP- Cancer care provider; PHC- Primary Healthcare Centre**)**

| **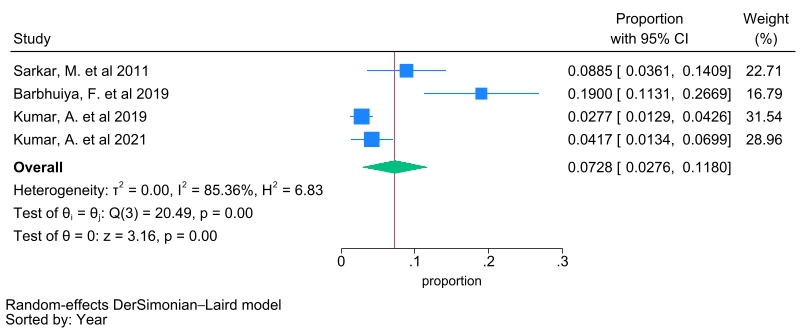** |
| --- |
| **Supplementary Figure 15. Forest plot showing proportion of individuals who first approached Quacks before reaching CCP**  Proportion from individual studies was calculated by dividing the number of individuals who initially approached quacks (unqualified treatment providers) for their cancer symptoms with total study population and synthesized by meta-analysis using random effect Dersimonian Laid model. (CCP- Cancer care provider; PHC- Primary Healthcare Centre**)** |

| **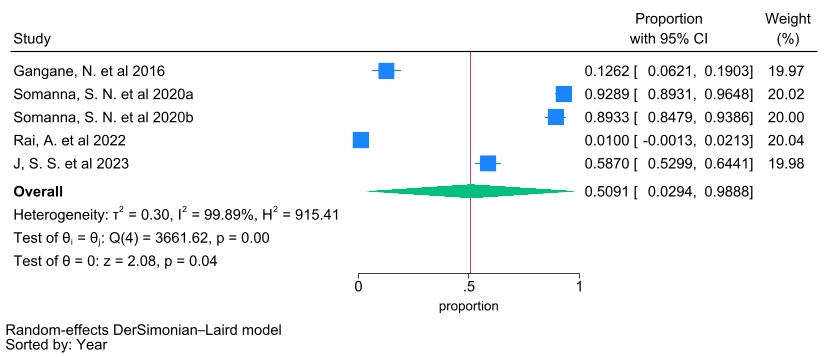** |
| --- |
| **Supplementary Figure 16. Forest plot showing ‘Lack of awareness’ as a reason for delay in seeking cancer care**  Proportion from individual studies was calculated by dividing the number of individuals who considered ‘Lack of awareness’ as a reason for delay in seeking cancer care with total number of individuals who experienced a delay, and synthesized by meta-analysis using random effect Dersimonian Laid model |
| **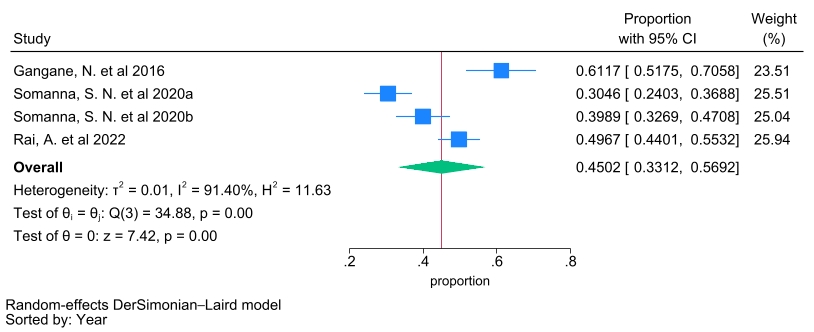** |
| **Supplementary Figure 17. Forest plot showing ‘Painlessness’ as a reason for delay in seeking cancer care** |

Proportion from individual studies was calculated by dividing the number of individuals who considered ‘Painlessness’ as a reason for delay in seeking cancer care with total number of individuals who experienced a delay, and synthesized by meta-analysis using random effect Dersimonian Laid model

| **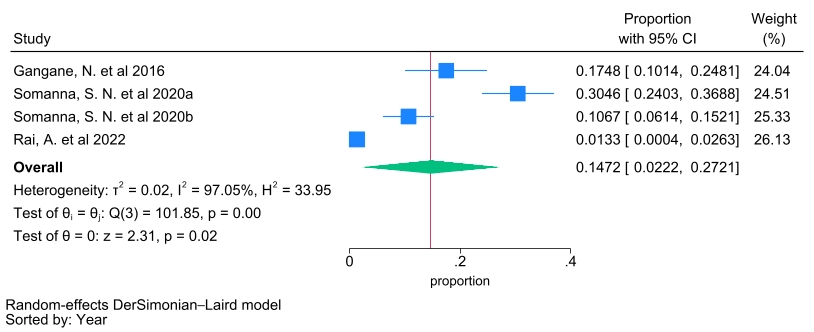** |
| --- |
| **Supplementary Figure 18. Forest plot showing ‘Financial constraints’ as a reason for delay in seeking cancer care** |

Proportion from individual studies was calculated by dividing the number of individuals who considered ‘Financial constraints’ as a reason for delay in seeking cancer care with total number of individuals who experienced a delay, and synthesized by meta-analysis using random effect Dersimonian Laid model

| **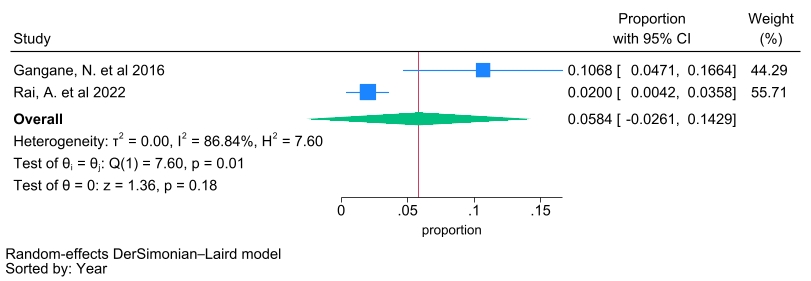** |
| --- |
| **Supplementary Figure 19. Forest plot showing ‘Fear of diagnosis’ as a reason for delay in seeking cancer care**  Proportion from individual studies was calculated by dividing the number of individuals who considered ‘Fear of diagnosis’ as a reason for delay in seeking cancer care with total number of individuals who experienced a delay, and synthesized by meta-analysis using random effect Dersimonian Laid model |

| **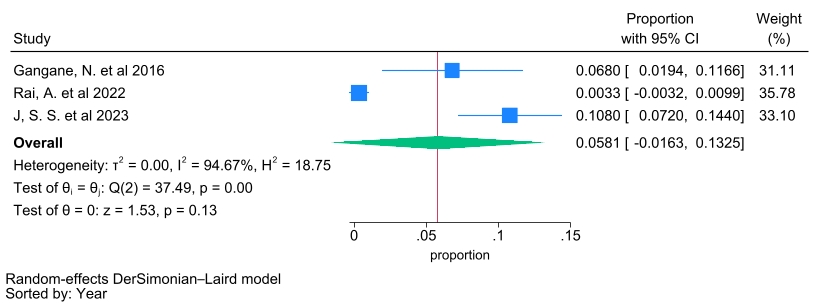** |
| --- |
| **Supplementary Figure 20. Forest plot showing ‘Family priorities’ as a reason for delay in seeking cancer care**  Proportion from individual studies was calculated by dividing the number of individuals who considered ‘Family priorities’ as a reason for delay in seeking cancer care with total number of individuals who experienced a delay, and synthesized by meta-analysis using random effect Dersimonian Laid model |
| **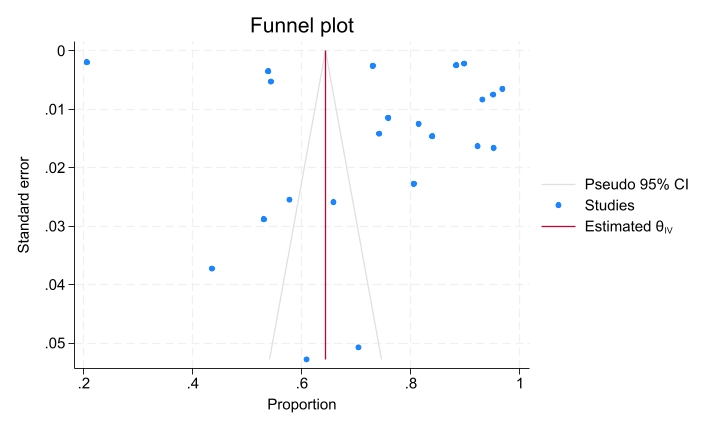** |
| **Supplementary Figure 21. Funnel plot of studies reporting proportion of individuals who sought treatment**  X axis represents proportion and y axis represents the precision of the measure (standard error). Studies reporting proportion of individuals who sought treatment are plotted as blue dots. The (two-sided) statistical significance of any point on a funnel plot can be calculated by considering the estimated measure and precision. |
| **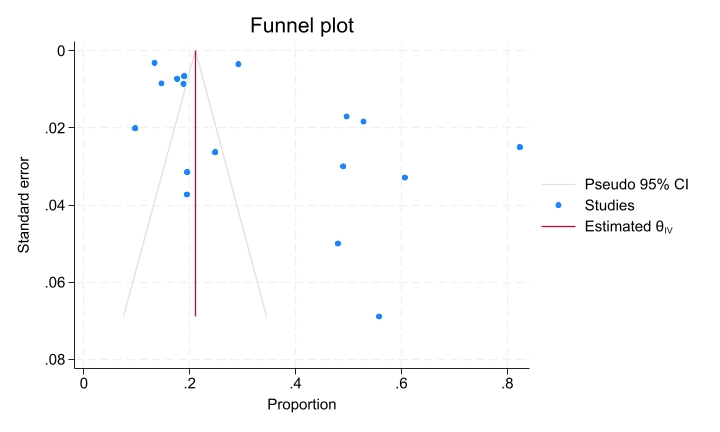** |
| **Supplementary Figure 22. Funnel plot of studies reporting proportion of individuals who visited Government facility for their illness**  X axis represents proportion and y axis represents the precision of the measure (standard error). Studies reporting proportion of individuals who sought treatment from Government facility are plotted as blue dots. The (two-sided) statistical significance of any point on a funnel plot can be calculated by considering the estimated measure and precision. |
| **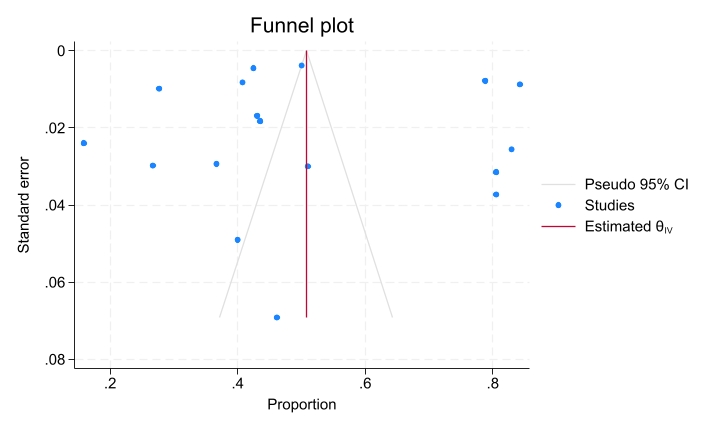** |
| **Supplementary Figure 23. Funnel plot of studies reporting proportion of individuals who visited Private facility for their illness**  X axis represents proportion and y axis represents the precision of the measure (standard error). Studies reporting proportion of individuals who sought treatment from Private facility are plotted as blue dots. The (two-sided) statistical significance of any point on a funnel plot can be calculated by considering the estimated measure and precision. |
| **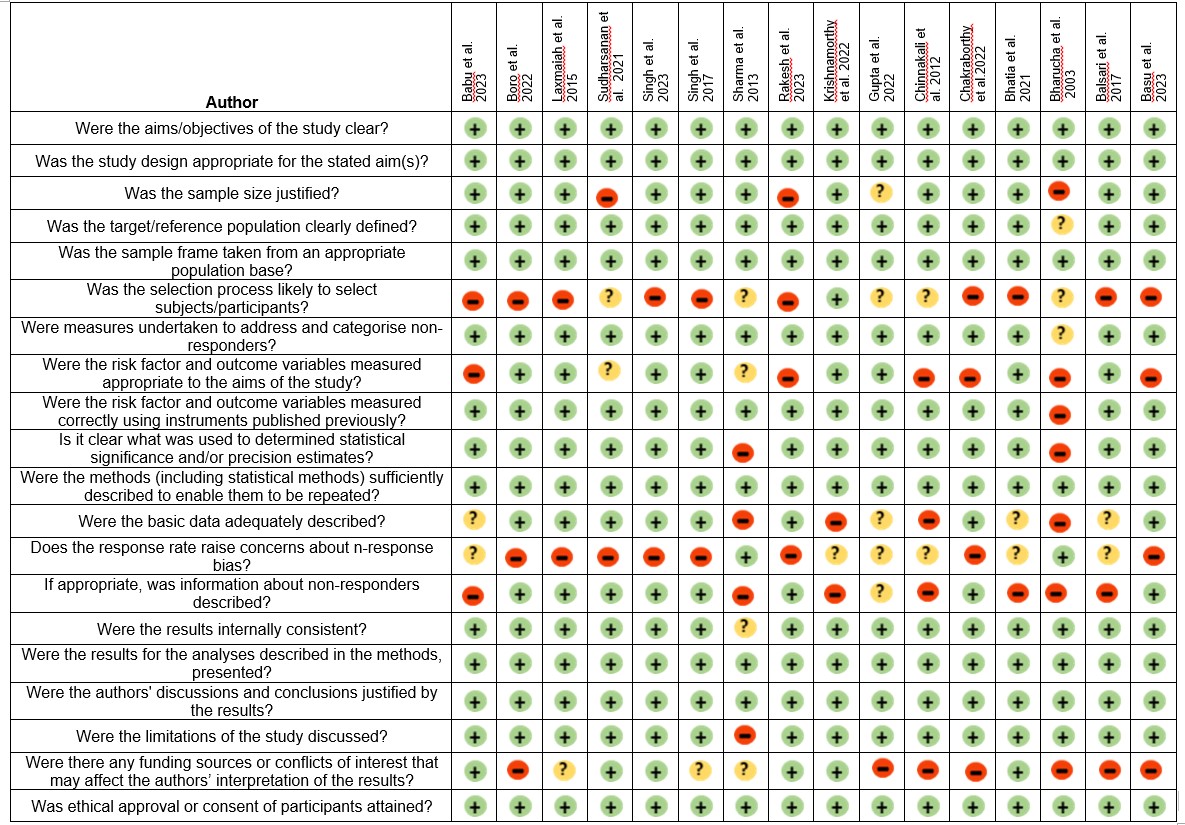**  *  * |
| **Supplementary Figure 24. Graphical reporting of risk of bias in studies reporting on treatment seeking for hypertension**  Figure showing risk of bias in studies reporting on treatment seeking for hypertension assessed using AXIS tool where ‘+’ symbol in green indicate YES (*No) and Low risk of bias, ‘?’ in yellow indicate Do not Know and ‘some concerns’ and ‘-‘symbol in red indicate NO (*Yes) and ‘serious concerns’ |

| **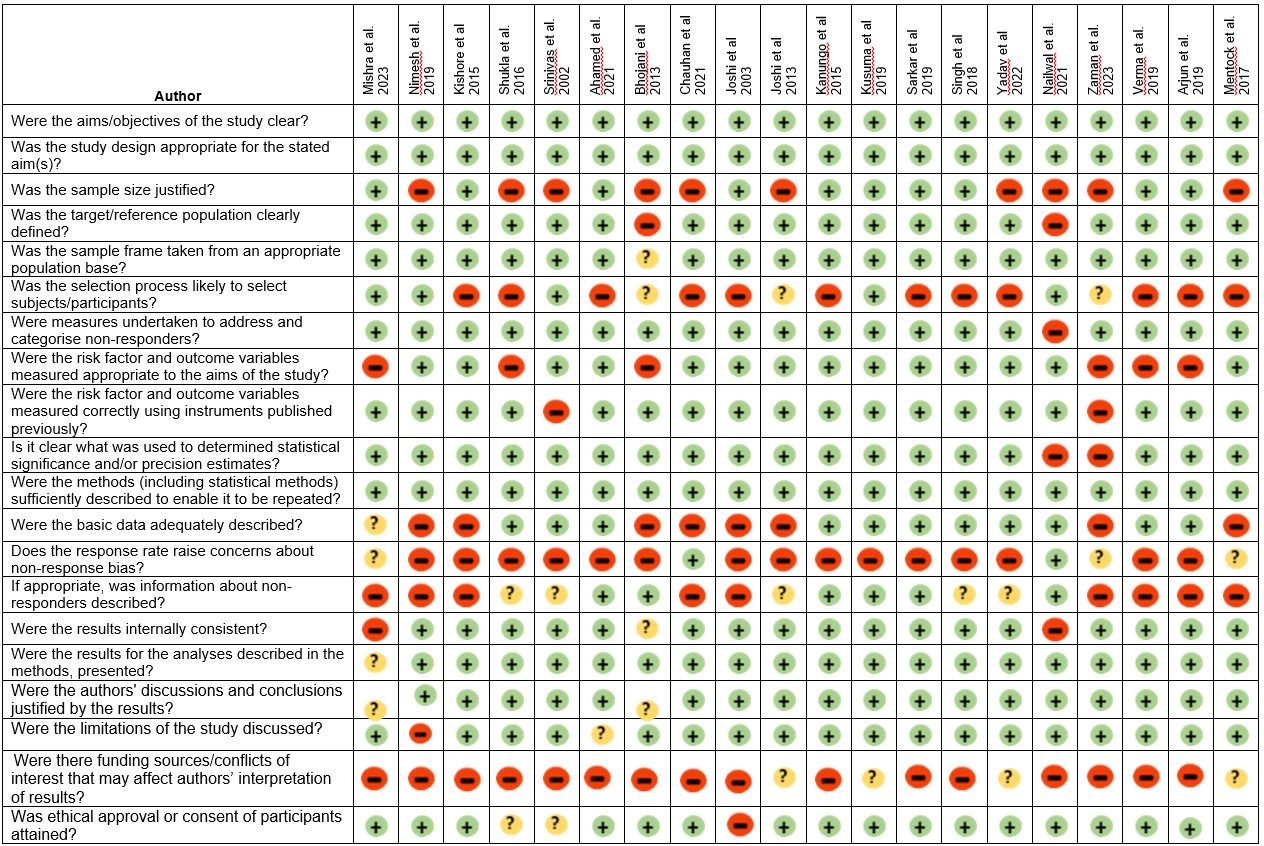**  *  * |
| --- |
| **Supplementary Figure 25. Graphical reporting of risk of bias in studies reporting on treatment seeking for diabetes and self-reported illnesses including NCDs**  Figure showing risk of bias in studies reporting on treatment seeking for diabetes and self-reported including NCDs assessed using AXIS tool where ‘+’ symbol in green indicate YES (*No) and Low risk of bias, ‘?’ in yellow indicate Do not Know and ‘some concerns’ and ‘-‘symbol in red indicate NO (*Yes) and ‘serious concerns’ |
| **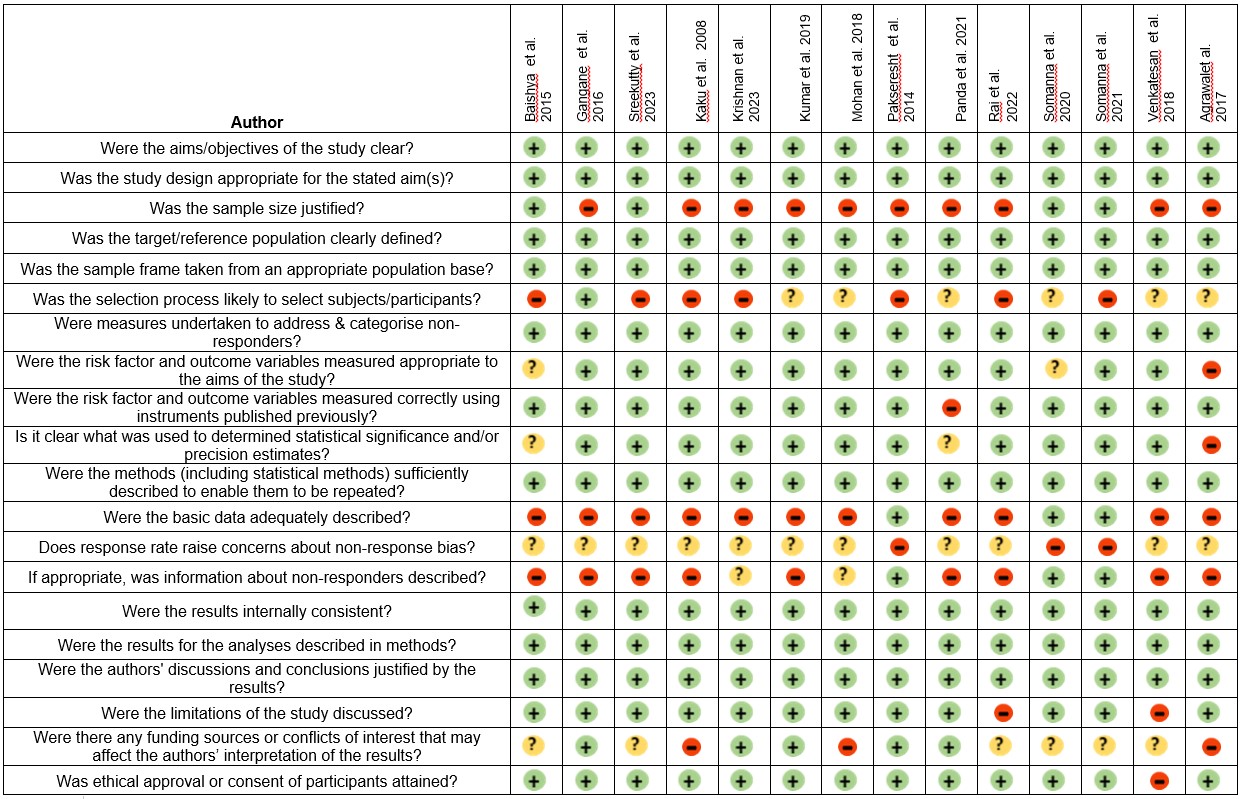**  *  * |
| **Supplementary Figure 26. Graphical reporting of risk of bias in studies reporting on presentation/treatment delay**  Figure showing risk of bias in studies reporting on treatment seeking for reporting on presentation/treatment delay assessed using AXIS tool where ‘+’ symbol in green indicate YES (*No) and Low risk of bias, ‘?’ in yellow indicate Do not Know and ‘some concerns’ and ‘-‘symbol in red indicate NO (*Yes) and ‘serious concerns’ |
| **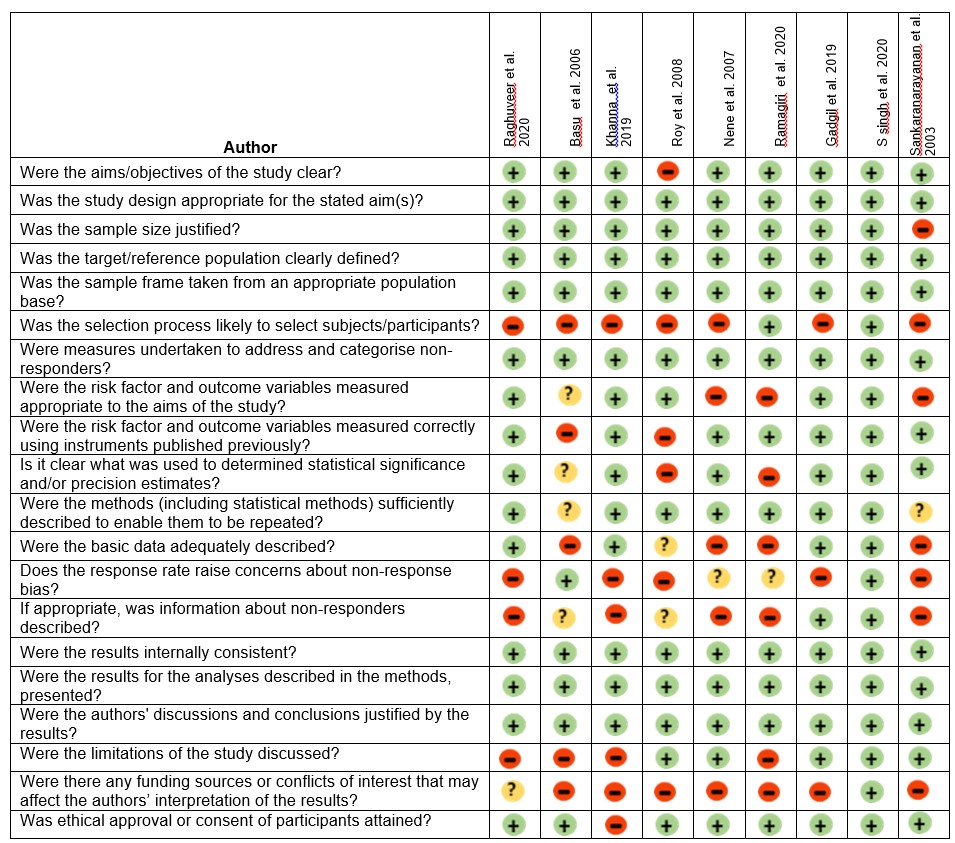**  *  * |
| **Supplementary Figure 27. Graphical reporting of risk of bias in studies reporting on screening uptake**  Figure showing risk of bias in studies reporting on treatment seeking for studies reporting on screening uptake assessed using AXIS tool where ‘+’ symbol in green indicate YES (*No) and Low risk of bias, ‘?’ in yellow indicate Do not Know and ‘some concerns’ and ‘-‘symbol in red indicate NO (*Yes) and ‘serious concerns’ |

| **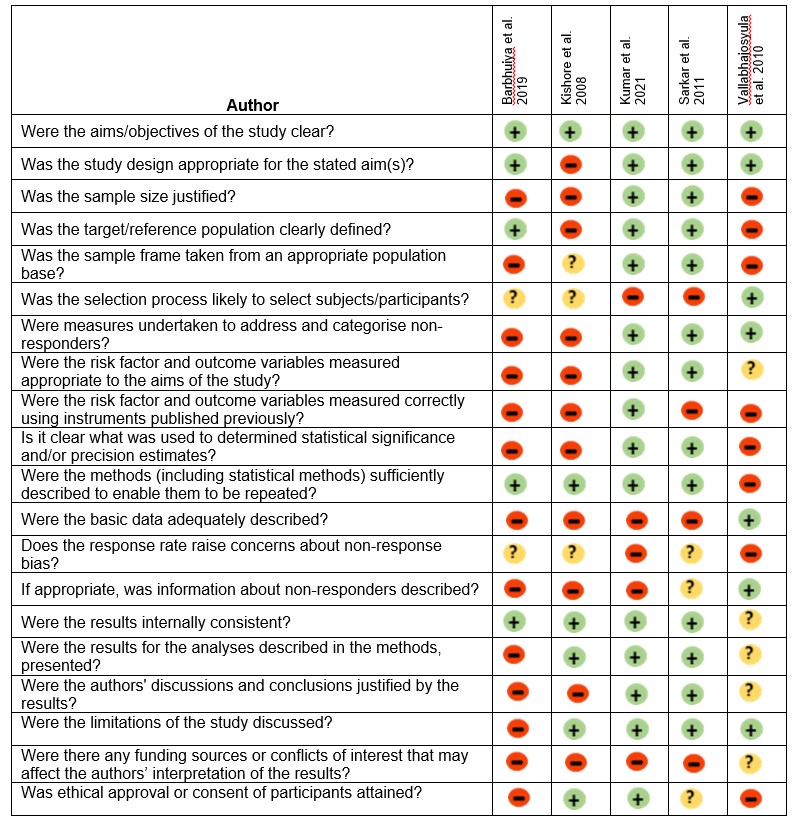**  *  * |
| --- |
| **Supplementary Figure 28. Graphical reporting of risk of bias in studies reporting on initial treatment trajectory for cancer**  X axis represents proportion and y axis represents the precision of the measure (standard error). Studies reporting proportion of individuals who sought treatment from Private facility are plotted as blue dots. The (two-sided) statistical significance of any point on a funnel plot can be calculated by considering the estimated measure and precision. |

**Appendix I**

PRISMA 2020 Abstract Checklist

| **Section and Topic** | **Item #** | **Checklist item** | **Reported (Yes/No)** |
| --- | --- | --- | --- |
| **TITLE** | | |  |
| Title | 1 | Identify the report as a systematic review. | 1 |
| **BACKGROUND** | | |  |
| Objectives | 2 | Provide an explicit statement of the main objective(s) or question(s) the review addresses. | 2 |
| **METHODS** | | |  |
| Eligibility criteria | 3 | Specify the inclusion and exclusion criteria for the review. | 2 |
| Information sources | 4 | Specify the information sources (e.g. databases, registers) used to identify studies and the date when each was last searched. | 2 |
| Risk of bias | 5 | Specify the methods used to assess risk of bias in the included studies. | 2 |
| Synthesis of results | 6 | Specify the methods used to present and synthesise results. | 2 |
| **RESULTS** | | |  |
| Included studies | 7 | Give the total number of included studies and participants and summarise relevant characteristics of studies. | 2 |
| Synthesis of results | 8 | Present results for main outcomes, preferably indicating the number of included studies and participants for each. If meta-analysis was done, report the summary estimate and confidence/credible interval. If comparing groups, indicate the direction of the effect (i.e. which group is favoured). | 2 |
| **DISCUSSION** | | |  |
| Limitations of evidence | 9 | Provide a brief summary of the limitations of the evidence included in the review (e.g. study risk of bias, inconsistency and imprecision). | 2 |
| Interpretation | 10 | Provide a general interpretation of the results and important implications. | 2 |
| **OTHER** | | |  |
| Funding | 11 | Specify the primary source of funding for the review. | 16 |
| Registration | 12 | Provide the register name and registration number. | 2 |

*From:*  Page MJ, McKenzie JE, Bossuyt PM, Boutron I, Hoffmann TC, Mulrow CD, et al. The PRISMA 2020 statement: an updated guideline for reporting systematic reviews. BMJ 2021;372:n71. doi: 10.1136/bmj.n71. This work is licensed under CC BY 4.0. To view a copy of this license, visit <https://creativecommons.org/licenses/by/4.0/>

**Appendix II**

PRISMA 2020 Statement

| **Section and Topic** | **Item #** | **Checklist item** | **Location where item is reported** |
| --- | --- | --- | --- |
| **TITLE** | | |  |
| Title | 1 | Identify the report as a systematic review. | 1 |
| **ABSTRACT** | | |  |
| Abstract | 2 | See the PRISMA 2020 for Abstracts checklist. | Appendix I |
| **INTRODUCTION** | | |  |
| Rationale | 3 | Describe the rationale for the review in the context of existing knowledge. | 1 |
| Objectives | 4 | Provide an explicit statement of the objective(s) or question(s) the review addresses. | 1 |
| **METHODS** | | |  |
| Eligibility criteria | 5 | Specify the inclusion and exclusion criteria for the review and how studies were grouped for the syntheses. | 2 |
| Information sources | 6 | Specify all databases, registers, websites, organisations, reference lists and other sources searched or consulted to identify studies. Specify the date when each source was last searched or consulted. | 2 |
| Search strategy | 7 | Present the full search strategies for all databases, registers and websites, including any filters and limits used. | Supp material 1 & 2 |
| Selection process | 8 | Specify the methods used to decide whether a study met the inclusion criteria of the review, including how many reviewers screened each record and each report retrieved, whether they worked independently, and if applicable, details of automation tools used in the process. | 2 |
| Data collection process | 9 | Specify the methods used to collect data from reports, including how many reviewers collected data from each report, whether they worked independently, any processes for obtaining or confirming data from study investigators, and if applicable, details of automation tools used in the process. | 2 |
| Data items | 10a | List and define all outcomes for which data were sought. Specify whether all results that were compatible with each outcome domain in each study were sought (e.g. for all measures, time points, analyses), and if not, the methods used to decide which results to collect. | 2 |
|  | 10b | List and define all other variables for which data were sought (e.g. participant and intervention characteristics, funding sources). Describe any assumptions made about any missing or unclear information. | 5 |
| Study risk of bias assessment | 11 | Specify the methods used to assess risk of bias in the included studies, including details of the tool(s) used, how many reviewers assessed each study and whether they worked independently, and if applicable, details of automation tools used in the process. | 3 |
| Effect measures | 12 | Specify for each outcome the effect measure(s) (e.g. risk ratio, mean difference) used in the synthesis or presentation of results. | 3 |
| Synthesis methods | 13a | Describe the processes used to decide which studies were eligible for each synthesis (e.g. tabulating the study intervention characteristics and comparing against the planned groups for each synthesis (item #5)). | Table 1 |
|  | 13b | Describe any methods required to prepare the data for presentation or synthesis, such as handling of missing summary statistics, or data conversions. | 3 |
|  | 13c | Describe any methods used to tabulate or visually display results of individual studies and syntheses. | 3 |
|  | 13d | Describe any methods used to synthesize results and provide a rationale for the choice(s). If meta-analysis was performed, describe the model(s), method(s) to identify the presence and extent of statistical heterogeneity, and software package(s) used. | 3 |
|  | 13e | Describe any methods used to explore possible causes of heterogeneity among study results (e.g. subgroup analysis, meta-regression). | 3 |
|  | 13f | Describe any sensitivity analyses conducted to assess robustness of the synthesized results. | 3 |
| Reporting bias assessment | 14 | Describe any methods used to assess risk of bias due to missing results in a synthesis (arising from reporting biases). | 3 |
| Certainty assessment | 15 | Describe any methods used to assess certainty (or confidence) in the body of evidence for an outcome. | - |
| **RESULTS** | | |  |
| Study selection | 16a | Describe the results of the search and selection process, from the number of records identified in the search to the number of studies included in the review, ideally using a flow diagram. | 6 |
|  | 16b | Cite studies that might appear to meet the inclusion criteria, but which were excluded, and explain why they were excluded. | 6 |
| Study characteristics | 17 | Cite each included study and present its characteristics. | Table 1 |
| Risk of bias in studies | 18 | Present assessments of risk of bias for each included study. | 6 |
| Results of individual studies | 19 | For all outcomes, present, for each study: (a) summary statistics for each group (where appropriate) and (b) an effect estimate and its precision (e.g. confidence/credible interval), ideally using structured tables or plots. | Figure 2 |
| Results of syntheses | 20a | For each synthesis, briefly summarise the characteristics and risk of bias among contributing studies. | 6 |
|  | 20b | Present results of all statistical syntheses conducted. If meta-analysis was done, present for each the summary estimate and its precision (e.g. confidence/credible interval) and measures of statistical heterogeneity. If comparing groups, describe the direction of the effect. | Figure 2,3 & Supplementary Figures |
|  | 20c | Present results of all investigations of possible causes of heterogeneity among study results. | 7-9 |
|  | 20d | Present results of all sensitivity analyses conducted to assess the robustness of the synthesized results. | 7-9 |
| Reporting biases | 21 | Present assessments of risk of bias due to missing results (arising from reporting biases) for each synthesis assessed. | 7-9 |
| Certainty of evidence | 22 | Present assessments of certainty (or confidence) in the body of evidence for each outcome assessed. | - |
| **DISCUSSION** | | |  |
| Discussion | 23a | Provide a general interpretation of the results in the context of other evidence. | 10&11 |
|  | 23b | Discuss any limitations of the evidence included in the review. | 10&11 |
|  | 23c | Discuss any limitations of the review processes used. | 10&11 |
|  | 23d | Discuss implications of the results for practice, policy, and future research. | 10&11 |
| **OTHER INFORMATION** | | |  |
| Registration and protocol | 24a | Provide registration information for the review, including the register name and registration number, or state that the review was not registered. | 4 |
|  | 24b | Indicate where the review protocol can be accessed, or state that a protocol was not prepared. | 4 |
|  | 24c | Describe and explain any amendments to information provided at registration or in the protocol. | - |
| Support | 25 | Describe sources of financial or non-financial support for the review, and the role of the funders or sponsors in the review. | 12 |
| Competing interests | 26 | Declare any competing interests of review authors. | 12 |
| Availability of data, code and other materials | 27 | Report which of the following are publicly available and where they can be found: template data collection forms; data extracted from included studies; data used for all analyses; analytic code; any other materials used in the review. | 11 |

*From:*  Page MJ, McKenzie JE, Bossuyt PM, Boutron I, Hoffmann TC, Mulrow CD, et al. The PRISMA 2020 statement: an updated guideline for reporting systematic reviews. BMJ 2021;372:n71. doi: 10.1136/bmj.n71
